# Heat-killed *Akkermansia muciniphila* ameliorates allergic airway inflammation in mice

**DOI:** 10.3389/fmicb.2024.1386428

**Published:** 2024-05-09

**Authors:** Seol Ah Yoon, Younggap Lim, Hye Rim Byeon, Jiyeon Jung, Seongho Ma, Moon-Gi Hong, Dohak Kim, Eun-Ji Song, Young-Do Nam, Jae-Gu Seo, Sang-Nam Lee

**Affiliations:** ^1^R&D Center, Enterobiome Inc., Ilsandong-gu, Goyang-si, Republic of Korea; ^2^Research Group of Personalized Diet, Korea Food Research Institute, Wanju-gun, Jeollabuk-do, Republic of Korea

**Keywords:** *Akkermansia muciniphila*, allergic asthma, heat-killed form, house dust mite, next generation probiotics

## Abstract

Allergic asthma (AA) is a common inflammatory airway disease characterized by increased airway hyper-responsiveness (AHR), inflammation, and remodeling. *Akkermansia muciniphila* is a strictly anaerobic bacterium residing in the gut and is a promising next-generation probiotic to improve metabolic inflammatory syndrome. A recent study suggested the beneficial effect of live *A. muciniphila* on allergic airway inflammation (AAI) in mice. However, whether the heat-killed form can improve AAI requires further investigation. Mice sensitized and challenged with house dust mites (HDM) develop AA hallmarks including inflammatory cell infiltration, goblet cell hyperplasia, and subepithelial collagen deposition in the lungs. These phenomena were reversed by oral administration of the heat-killed *A. muciniphila* strain EB-AMDK19 (AMDK19-HK) isolated from the feces of healthy Koreans. Furthermore, AMDK19-HK diminished the HDM-induced AHR to inhaled methacholine, lung mast cell accumulation, and serum HDM-specific IgE levels. It also led to the overall suppression of IL-4, IL-13, and eotaxin production in bronchoalveolar lavage fluids, and *Il4*, *Il5*, *Il13*, and *Ccl17* gene expression in lung tissues. Moreover, AMDK19-HK suppressed Th2-associated cytokine production in the splenocytes of HDM-sensitized mice *in vitro*. Additionally, a combination of *16S rRNA* gene sequencing and short-chain fatty acid (SCFA) analysis in cecal samples revealed that AMDK19-HK modulated the relative abundance of circulating SCFA-associated gut genera, including a positive correlation with *Lachnospiraceae_ NK4A136_group* and a negative correlation with *Lachnoclostridium* and significantly increased cecal SCFA concentrations. Finally, AMDK19-HK improved intestinal mucosal barrier function. These results suggest that the oral administration of AMDK19-HK ameliorates HDM-induced AAI in mice by suppressing Th2-mediated immune responses and could have a protective effect against AA development.

## 1 Introduction

Asthma is a heterogeneous disease with various clinical phenotypes, including disease severity, obesity, inflammatory pathways, age at onset, and corticosteroid responsiveness (Yang et al., 2021). Thus, asthma symptoms are difficult to control with pharmacologic management due to the inter-individual variability in response to asthma medications, including β2-adrenergic agonists, anticholinergics, corticosteroids, leukotriene modifiers, and biologics (Fanta, 2009; Lambrecht and Hammad, 2015). Therefore, personalized asthma management treatments based on variable clinical presentations (phenotypes) and distinct mechanistic pathways (endotypes) must be developed. Asthma is divided into two major groups, type2 (T2)-high (eosinophilic) and T2-low (neutrophilic), based on the presence or absence of T-helper (Th2) cytokines (IL-4, IL-5, and IL-13) and eosinophils in the blood and tissues (Kuruvilla et al., 2019). Allergic asthma (AA) is an eosinophilic T2-high immune disease caused by aeroallergens, such as dust mites, pollen, and pet dander. It is characterized by high levels of serum IgE and the activation of immune cells such as Th2 cells, eosinophils, mast cells, basophils, innate lymphoid cells (ILCs), and dendritic cells (DCs; Kubo, 2017; Caminati et al., 2018). Furthermore, AA is closely associated with airway hyperresponsiveness (AHR), airway inflammation, mucus overproduction due to goblet cell hyperplasia, and airway remodeling (Kubo, 2017; Caminati et al., 2018; Yang et al., 2021). In the immune system, Th1-driven cytokines including IFN-γ and IL-12 restrict the proliferation of Th2 cells and thus a shift in the Th1/Th2 balance towards Th2 dominance is key in the onset of AA (Luo et al., 2022). Moreover, IL-17-producing Th17 cells augment Th2 responses and IL-10–producing Treg cells suppress Th2 responses; thus, a shift in Th17/regulatory T cells (Tregs) towards Th17 dominance also plays an important role in the pathogenesis of AA (Verstegen et al., 2021).

The gut microbiota directly or indirectly affects multiple physiological functions of the host, including nutrition, metabolism, and immunity through multidirectional and dynamic interactions between the gut and distant organs (Wang et al., 2017; Ghosh et al., 2021; Verstegen et al., 2021). For instance, circulating bioactive metabolites produced by gut microbiota, such as short-chain fatty acids (SCFAs) can modulate Th2 immunity via a bidirectional crosstalk between the gut microbiota and the lungs, termed the “microbiota-gut-lung axis” (Verstegen et al., 2021). Thus, probiotic research is focused on developing the human gut microbiota as possible probiotic agents for the prevention and treatment of dysbiosis-associated diseases. These potentially beneficial gut microbes are termed next-generation probiotics (NGP; Martín and Langella, 2019). *Akkermansia muciniphila* is an NGP candidate with promising applications; it is a gram-negative and strict anaerobe belonging to the phylum *Verrucomicrobia* comprising 1–3% of the healthy adult gut microbiota (Derrien et al., 2004, 2008; Cani et al., 2022). This bacterium grows with mucin, which is the main component of the mucus layer covering the epithelium, and produces SCFAs (mainly acetate and propionate) and amino acid metabolites in the host (Derrien et al., 2004; Liu et al., 2022). Additionally, *A. muciniphila* plays a critical role in maintaining balanced gut microbial communities and gut barrier function, and preventing endotoxin-induced systemic inflammation (Liu et al., 2021). Reduced levels of *A. muciniphila* have been shown to be associated with metabolic inflammatory diseases such as obesity, type 2 diabetes, nonalcoholic fatty liver disease, and hypertension (Cani et al., 2022), and immune-related inflammatory diseases such as psoriasis, atopic dermatitis, asthma, and cancer (Demirci et al., 2019; Effendi et al., 2022). Moreover, oral administration of *A. muciniphila* improves metabolic and immune-related diseases by enhancing gut barrier function, the host immune system, and metabolic processes (Cani et al., 2022; Effendi et al., 2022). However, a precise description of the molecular mechanisms underlying the beneficial effects of *A. muciniphila* is lacking.

Although adequate intake of NGP is beneficial to the host, technological limitations remain in maintaining its stability and viability owing to its high sensitivity to oxygen (Siciliano et al., 2021). Thus, there is an increased interest in developing novel nutraceuticals based on inactivated probiotics that are safer and more stable and are termed paraprobiotics (Siciliano et al., 2021). Preclinical and clinical studies demonstrated that supplementing mice with a high fat diet (HFD) plus *A. muciniphila* killed by pasteurization at 70°C for 30 min could counteract the HFD-induced obesity-related metabolic symptoms, with no issues about safety or tolerability (Plovier et al., 2017; Depommier et al., 2019). Therefore, the European Food Safety Authority has declared pasteurized *A. muciniphila* a novel food ingredient (EFSA Panel on Nutrition, Novel Foods and Food Allergens (NDA) et al., 2021). Recently, Michalovich et al. demonstrated a negative correlation between fecal *A. muciniphila* levels and asthma severity in human subjects (Michalovich et al., 2019). They also showed that supplementation of live *A. muciniphila*, but not its heat-killed form inactivated by boiling at 100°C for 15 min, markedly alleviated airway inflammation in allergen-induced AA mouse model, thereby suggesting that the cell-derived components for the protective effect is sensitive to boiling. However, whether heat-killed *A. muciniphila* inactivated by pasteurization can improve allergic airway inflammation (AAI) has not yet been evaluated. In this study, we investigated the anti-allergic and anti-inflammatory effects of the heat-killed (pasteurized) *A. muciniphila* strain EB-AMDK19 (AMDK19-HK) isolated from healthy Korean human feces during AAI. Using a mouse model of AA induced by house dust mite (HDM) extract, a major perennial allergen associated with human asthma, we found that the oral administration of AMDK19-HK alleviated AAI by suppressing airway goblet cell hyperplasia, subepithelial collagen deposition, eosinophilia, Th2-associated cytokine and chemokine production, AHR to methacholine, and serum IgE production. Moreover, AMDK19-HK significantly decreased the production of Th2-associated cytokines in splenocytes of HDM-sensitized mice upon *in vitro* HDM restimulation. Finally, AMDK19-HK administration modulated the relative abundance of specific SCFA-associated cecal microbiota, cecal levels of SCFAs, and enhanced intestinal mucosal barrier function. These results demonstrated that AMDK19-HK suppresses Th2-mediated inflammatory responses in the airways.

## 2 Materials and methods

### 2.1 Preparation of *Akkermansia muciniphila* EB-AMDK19-HK

*Akkermansia muciniphila* EB-AMDK19 (AMDK19, KCTC13761BP) was isolated from the fecal samples of healthy Korean subjects, as described in our previous report (Yang et al., 2020). The fecal samples were collected with approval from the Public Institution Bioethics Committee under the Ministry of Health and Welfare, South Korea (Approval no. P01-201705-31-002). AMDK19 was isolated by dilution to extinction of feces in anaerobic culture medium containing mucin as energy source, 0.4 g/L KH_2_PO_4_, 0.53 g/L Na_2_HPO_4_, 0.3 g/L NH_4_Cl, 0.3 g/L NaCl; 0.1 g/L MgCl_2_.6H_2_O, 0.11 g/L CaCl_2_, 1 mL alkaline and acid trace element solutions, 1 mL vitamin solution, 4 g/L NaHCO_3_, 0.25 g/L Na_2_S.9H_2_O, and 0.25 g/L hog gastric mucin (Type III, Sigma-Aldrich, Saint Louis, MO, United States) as described previously (Derrien et al., 2004). Strain identification was confirmed by 16S ribosomal RNA (rRNA) gene sequencing. For the preparation of AMDK19, the strain was anaerobically cultured in a soy-peptone-based medium containing 20 g/L soy-peptone, 10 g/L yeast extract, 2.5 g/L K2HPO4, 5 g/L N-acetyl-D-glucosamine, 2.5 g/L D-fructose, 5 g/L D-lactose, 8 g/L L-aspartic acid, 0.1 mg/L cyanocobalamin, and 0.5 g/L L-cysteine hydrochloride at 37°C until the early stationary phase. Cultures were inactivated by pasteurization for 70°C for 30 min. The pasteurized cells were collected using tangential flow filtration systems (Sartorius Stedim Biotech, Göttingen, Germany) and freeze-dried in an industrial apparatus FDT-8650 (Operon Co., Ltd., Gyeonggi-do, Korea) under the following conditions: freezing at −80°C, primary drying at −30°C under vacuum, and secondary drying at 20°C under vacuum. The lyophilized powder was stored at −80°C until use. Before administration by oral gavage, lyophilized bacteria were resuspended in phosphate-buffered saline (PBS) and counted using a hemocytometer under a microscope. The center square millimeter (1 mm × 1 mm squares) is subdivided into 25 small squares and the cell-depth is 0.1 mm, so that the volume over a small square (0.04 mm^2^) is 0.004 mm^3^ (1 mm^3^ is equal to 1 μL). Cells were counted within four small squares, and the results were averaged. Number of cells per milliliter (mL) = number of cells counted × dilution factor × 250,000.

### 2.2 Mice

Five-week-old male C57BL/6 mice were purchased from Orient Bio, Inc. (Seongnam-si, South Korea). All mice were housed under a constant light/dark cycle under specific pathogen-free conditions. All procedures followed the guidelines of the International Association for the Study of Pain policies on the use of laboratory animals and were approved by the Ethics Committee and Institutional Animal Care and Use Committee of the Dongguk University Health System (IACUC-2022-029-1).

### 2.3 Generation of AA mouse model

**Figure 1:**
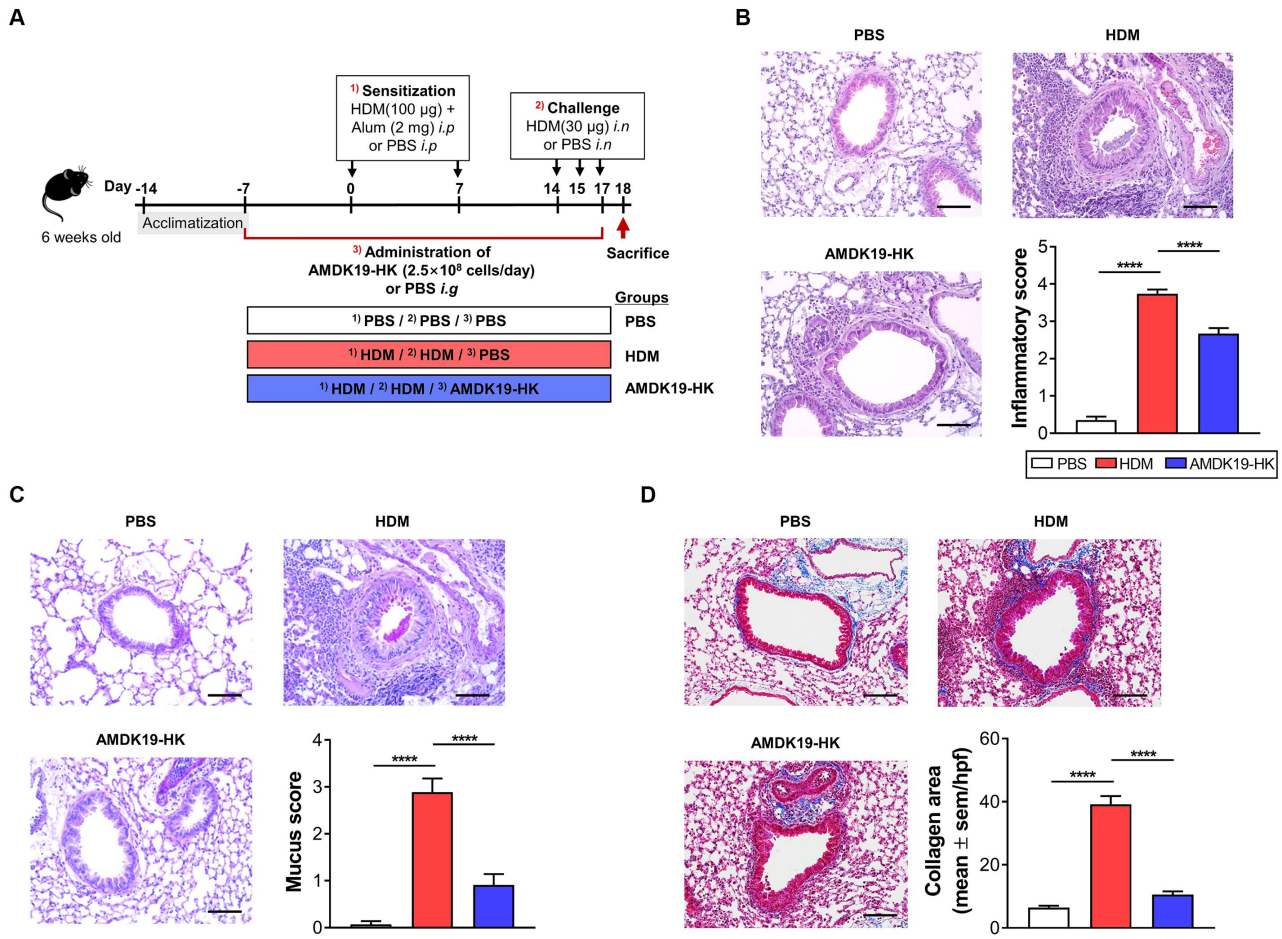
Effects of AMDK19-HK on airway inflammation and remodeling in HDM-induced AA model. **(A)** Experimental design of the AA study on mice. A mouse model of AA mice was established by sensitization with HDM extracts and consequent challenge with HDM. AMDK19-HK was prepared by pasteurization for 30 min at 70°C. The AMDK19-HK group were given AMDK19-HK (2.5 × 10^8^ cells/day) via oral gavage from 1 week before the first sensitization until the end of the experiment. Mice treated with PBS under the same experimental condition were used as an untreated control. On the end day of the experiment, lungs were collected, fixed, embedded in paraffin, and sectioned at 5-μm thickness. **(B)** Representative images of H&E-stained lung sections. Scale bar, 100 μm. For semi-quantification, inflammatory cell infiltration was scored based on 5-point scale (0–4). **(C)** Representative images of PAS-stained lung sections. For semi-quantification, distribution of goblet cells and mucus production were scored based on 5-point scale (0–4). **(D)** Representative images of M-T-stained lung sections. Blue regions indicate collagen deposition. Semi-quantification of collagen deposition was performed by ImageJ software. Data are expressed as mean ± SEM from a representative experiment of 3 performed (*n* = 5–6 mice per group). ^****^p < 0.0001, Mann–Whitney *U*-test. i.p, intraperitoneal sensitization; i.n, intranasal challenge; i.g, intragastric injection; AA, allergic asthma, H&E, hematoxylin and eosin; HDM, house dust mite; PAS, periodic acid-Schiff; M-T, Masson’s trichrome.

The AA mouse model was created according to a previous protocol with some modifications (Figure 1A; Ryu et al., 2013). After 1 week of acclimation, mice were randomly divided into five groups (each group *n* = 6–8) as follows: normal control mice (PBS, as control); HDM-induced AA mice (HDM); HDM mice supplemented with AMDK19-HK (AMDK19-HK); and HDM mice supplemented with dexamethasone (DEXA). Mice in the HDM, AMDK19-HK, and DEXA groups were sensitized by intraperitoneal injection with 100 μg of HDM extracts (Greer Laboratories, Lenoir, NC, United States) supplemented with 2 mg of aluminum hydroxide (Serva, Heidelberg, Germany) in 200 μL of PBS on days 0 and 7. One week after the last sensitization injection, mice were challenged intranasally with 30 μg of HDM extracts for 3 consecutive days under anesthesia with Zoletil^®^ (tiletamine-zolazepam, Virbac, Carros, France) and Rompun^®^ (xylazine-hydrochloride, Bayer, Leverkusen, Germany), each at a dose of 1 mL/kg. The PBS group was sensitized and challenged with PBS/alum and PBS. The DEXA group received an intragastric injection of 2 mg/kg/day of DEXA 1 h before the HDM challenge. The AMDK19-HK group were given AMDK19-HK via oral gavage from 1 week before the first sensitization until the end of the experiment once a day. Serial dilution was performed to prepare 2.5 × 10^6^ to 2.5 × 10^9^ cells/100 μL of PBS per mouse. Other experimental groups received only PBS. Mice were sacrificed 24 h after the last challenge (day 18).

### 2.4 Samples collection

Blood samples were collected into anticoagulant-free tubes by cardiac puncture and fractionated by centrifugation at 2,000 ×*g* for 15 min at 4°C. Serum layers were taken out separately and stored at −80°C for IgE, cytokine and chemokine analysis. BALF was isolated in 0.8 mL of sterile PBS with 1x Protease Inhibitor Cocktail (Roche, Basel, Switzerland) and the differential cell counts in BALFs were determined by staining cytospin preparations (Shandon Inc., Pittsburgh, PA, United States) with Diff-Quik (IMEB Inc., San Marcos, CA, United States). Then, the remaining BALFs were centrifuged at 600 ×*g* for 10 min at 4°C and the supernatants were stored at −80°C for cytokine and chemokine analysis. The lung and spleen were taken for flow cytometric analysis and *in vitro* experiments. A part of the lung and intestine (jejunum, ileum, colon) were fixed in 4% paraformaldehyde for histology analysis, and the other parts were stored at −80°C for real-time reverse transcription polymerase chain reaction (RT-qPCR) analysis. The cecal contents were collected and stored at −80°C for further analyses of the SCFAs and microbiota.

### 2.5 Histology and immunohistochemistry

Formalin-fixed lung sections were embedded in paraffin, sectioned (4 μm), and stained with hematoxylin and eosin (H&E), periodic acid-Schiff (PAS), Alcian Blue/PAS (Abcam, Cambridge, MA, United States), Masson’s trichrome (Sigma-Aldrich), or toluidine blue (Sigma; 89,640-5G) via standard protocols. Histopathological scores were obtained in a blinded manner using a Nikon Eclipse Ni microscope (Nikon, Tokyo, Japan). Inflammatory cell infiltration and goblet cell distribution around the airway were evaluated using a semiquantitative scoring system (Yoshioka et al., 2009). To quantify inflammatory cells, a 5-point grading scale was used: 0, no infiltration; 1, few cells; 2, a ring of inflammatory cells of one cell layer deep; 3, a ring of inflammatory cells of two to four cells deep; and 4, a ring of inflammatory cells of more than four cells deep. To quantify goblet cells, a 5-point grading scale was used: 0, <0.5% PAS-positive cells; 1, <25%; 2, 25–50%; 3, 50–75%; and 4, >75%. The presence of collagen fibril deposition (calculations based on the airway basement membrane perimeter) and the total number of mast cells (cells in high-power fields per mm^2^ of lung parenchyma) were analyzed using ImageJ software. Eight fields were counted for each section, and the mean score from four to six mice per group was calculated.

For immunohistochemical staining, dewaxed sections were subjected to antigen retrieval by autoclaving in 10 mM sodium citrate buffer (pH 6.0). Endogenous peroxidase activity was quenched using 3% hydrogen peroxide for 10 min at room temperature. Nonspecific binding was blocked by incubation with a blocking buffer containing 2% bovine serum albumin for 30 min at room temperature. The primary Rabbit Anti-ZO-1 antibody (1:100, Cat# 61-7300, Invitrogen, Gaithersburg, MD, United States) was applied at room temperature for 1 h. Following washing in TBS, sections were incubated with labelled Polymer-horseradish peroxidase (EnVision+®, DAKO, Denmark) for 30 min at room temperature. After washing off the excess secondary antibody, the sections were developed with diaminobenzidine (DAB) solution (DAKO, Denmark) and counterstained with hematoxylin (Abcam, Cambridge, MA, United States). Staining was visualized using Nikon Eclipse Ni (Nikon, Japan) with Nikon NIS-Elements imaging software.

### 2.6 Measurement of antibodies, cytokines, chemokines, and SCFAs

Total IgE (BioLegend, San Diego, CA, United States), HDM-specific IgE (Chondrex, Redmond, WA, United States), cytokines (IL-4, IL-5, IL-13, IL-17A, and IL-6; BD Pharmingen, San Diego, CA, United States), and eotaxin (R&D Systems, Minneapolis, MN, United States) were analyzed using enzyme-linked immunosorbent assay (ELISA) kits according to the manufacturer’s instructions. SCFA levels in the cecal supernatants were analyzed using mass spectrometry (EZmass Co., Ltd., Gyeongnam, Korea). Briefly, 50 mg of cecal content were mixed with 500 μL of distilled water and 10 μL of 5 M HCl, sonicated for 10 min, and then added 400 μL of ether. The mixture was centrifuged, and 200 μL of supernatants were derivatized by adding 20 μL N,O-bis(trimethylsilyl)-trifluoroacetamide (BSTFA; Machery-Nagel, Düren, Germany). One microliter of the sample was injected into a GC-2010 Plus instrument equipped with a triple quadrupole mass spectrometer TQ-8030 (Shimadzu, Kyoto, Japan).

### 2.7 RNA isolation and RT-qPCR analysis

Total RNA from tissues and cultured cells was isolated with TRIzol reagent (Invitrogen, Gaithersburg, MD, United States) and reverse-transcribed into cDNA using M-MLV Reverse Transcriptase Kit (Thermo Fisher Scientific, Carlsbad, CA, United States). Reverse transcription-quantitative real-time polymerase chain reaction (RT-qPCR) analysis was performed using a 7,300 Real-Time PCR System (Applied Biosystems, Foster City, CA) and KAPA SYBR® FAST qPCR Kit (Roche, Indianapolis, IN, United States). Relative gene expression levels were evaluated after normalization to *Actb* or *Gapdh* mRNA expression using the ΔCt method. All primer sequences used in this study were shown in Supplementary Table S1.

### 2.8 Measurement of AHR

Twenty-four hours after the final HDM challenge, AHR was measured by whole-body plethysmography (Buxco Electronic Inc., Sharon, CT, United States) as previously described (Weckmann et al., 2007). Each mouse (*n* = 6 mice per group) was placed in the chamber for a 5-min acclimation period and the baseline readings were taken and averaged for 2 min. Aerosolized methacholine in increasing concentrations (0, 6.25, 12.5, 25 and 50 mg/mL PBS) were nebulized into the inhalation chamber for 2 min and readings were taken and averaged for 2 min after each nebulization. Enhanced pause (Penh) was determined and used as a measure of airway responsiveness to methacholine.

### 2.9 Flow cytometry

Lungs were treated with DNase I (Roche, Basel, Switzerland) and Dispase (Roche) for 30 min at 37°C and filtered through a 70 μm cell strainer (SPL Life Sciences, Gyeonggi-do, Korea) to generate single-cell suspensions. The LIVE/DEAD™ Fixable Dead Cell Stain was used prior to staining cells with the antibody master mix. Single cell suspensions of BALF, lung, and spleen cells were pre-incubated with Fc-receptor blocker (TruStain FcX™ PLUS) and then stained with fluorophore-labeled antibodies for specific cellular markers. A complete description of all antibodies is given in Supplementary Table S2. Flow cytometry data were acquired on a LSRFortessa flow cytometer (BD Biosciences) and then analyzed with FlowJo software (Tree star).

### 2.10 *In vitro* and *ex vivo* experiments with HDM-stimulated spleen cells

In the *in vitro* experiments, mice were sensitized with HDM/alum on days 0 and 7, and sacrificed on day 14. Spleens were aseptically recovered. Single-cell suspensions were prepared by gently teasing apart the tissue in RPMI1640 medium supplemented with10% heat-inactivated FBS (Thermo Fisher Scientific), 2 mM L-glutamine (Thermo Fisher Scientific), 10 mM HEPES (Thermo Fisher Scientific), 50 μM 2-mercaptoethanol (Sigma-Aldrich), 100 U/mL penicillin–streptomycin (Thermo Fisher Scientific), and then passed through a 70-μm cell strainer (SPL Life Sciences). Red blood cells were lysed using ammonium–chloride–potassium (ACK) lysis buffer (Thermo Fisher Scientific, Waltham, MA, United States). Single-cell suspensions from spleen (splenocytes) were cultured at 1 × 10^6^ cells/well in U-bottom 96-well plates in the presence of HDM (35 μg/mL) ± AMDK19-HK for 3 days. In the *ex vitro* experiments, mice were sensitized with HDM/alum on days 0 and 7, and AMDK19-HK was administered from 1 week before the first sensitization until the 1 day before the sacrifice. On day 14 splenocytes were obtained and restimulated with or without HDM for 3 days. Cytokine production was measured in supernatants by using ELISAs.

### 2.11 DNA extraction and *16S rRNA* gene amplicon sequencing

Bacterial community analysis was performed with the 16S rRNA gene sequencing method. In brief, DNA was extracted from cecal contents using the QIAamp^®^ DNA Stool Mini Kit (Qiagen, Germantown, MD, United States) according to manufacturer’s instructions. Bacterial 16S rRNA gene V1-V2 variable regions was amplified by PCR using unique 10-base barcoded primers. Amplicons were purified and sequenced on the Ion Torrent PGM system (Thermo Scientific, United States). Sequence analysis was performed using the Quantitative Insights into Microbial Ecology 2 (QIIME2, v2020.08). Quality-filtered raw sequence reads were classified into amplicon sequence variants (ASV) using the SILVA v138 database with a 99% identity threshold. Alpha and beta diversity analysis was performed using the QIIME2 diversity command. Permutational Multivariate Analysis of Variance (PERMANOVA) with the function adonis was used to detect differences in community composition between groups. Linear discriminant analysis (LDA) effect size (LEfSe) was performed to detect differentially abundant taxa in comparison of two groups, with LDA score (log10) over 2.0 and an alpha value of 0.05 for the factorial Kruskal-Wallis test. Correlation analysis was performed using Spearman correlation analysis. All raw sequencing data presented in this study were deposited in the Sequence Read Archive database under accession number PRJNA1074002.^1^

### 2.12 Statistical analysis

Statistical analysis was performed with GraphPad Prism 8.0.1 (GraphPad Software Inc., San Diego, CA, United States). All data are expressed as mean ± SEM or median (range). Differences between groups were calculated using Student’s *t*-test or Mann–Whitney *U*-test for the comparison of two groups (single variable), one-way ANOVA with Tukey post-test or Kruskal–Wallis test for single-variable comparisons with more than two groups, and two-way ANOVA with Bonferroni posttest for multi-variable analyses. Values of *p* < 0.05 were considered statistically significant.

## 3 Results

### 3.1 AMDK19-HK attenuates HDM-induced histopathological changes of allergic airway inflammation in mice

To investigate the influence of AMDK19-HK on airway remodeling due to AAI in the lung, mice were administered AMDK19-HK (2.5 × 10^8^ cells/day) or PBS (vehicle) from 1 week before the first sensitization with HDM until the end of the experiment via oral gavage (Figure 1A). Histopathological analysis of lung tissues was performed using hematoxylin and eosin (H&E) staining to assess inflammatory cell infiltration, periodic acid-Schiff (PAS) staining to detect mucus-producing goblet cells, and Masson’s trichrome staining to detect subepithelial collagen deposition. Massive infiltration of peribronchial inflammatory cells (Figure 1B) and hyperplasia of PAS-positive goblet cells (Figure 1C) were observed in the HDM group compared with the PBS group. However, these effects were attenuated by the administration of AMDK19-HK, as assessed by semi-quantitative scoring of H&E and PAS staining (Figures 1B,C). Masson’s trichrome staining also showed that subepithelial collagen deposition was significantly increased in the HDM group compared to that in the PBS group, whereas HDM-induced collagen deposition was decreased in the AMDK19-HK group (*p* < 0.0001; Figure 1D). Furthermore, we evaluated the dose–response effect of administration with AMDK19-HK in the concentration of 2.5 × 10^6^, 2.5 × 10^7^, 2.5 × 10^8^, or 2.5 × 10^9^ cells/day on HDM-induced airway inflammation and remodeling, including peribronchial cellular recruitment, goblet cell hyperplasia, and collagen deposition (Supplementary Figures S1A–S1D). Dexamethasone (DEXA), a potent inhibitor of airway inflammation and remodeling in asthma (Kumar et al., 2003), was used as the positive control. As expected, DEXA administration significantly attenuated the HDM-induced airway remodeling (Supplementary Figures S1B–S1D). HDM-induced airway inflammation and remodeling were significantly reduced by increasing the AMDK19-HK dose. Notably, a daily dose equal to or larger than 2.5 × 10^8^ cells/day showed a discernible useful effect on airway remodeling (*p* < 0.001), and there was no significant difference between the two high-doses (2.5 × 10^8^ and 2.5 × 10^9^ cells/day). Thereafter, all experiments were performed with an effective dose of 2.5 × 10^8^ cells. Collectively, these results suggest that AMDK19-HK attenuates AAI in an HDM-induced AA mouse model.

### 3.2 AMDK19-HK reduces HDM-induced airway hyperreactivity

**Figure 2:**
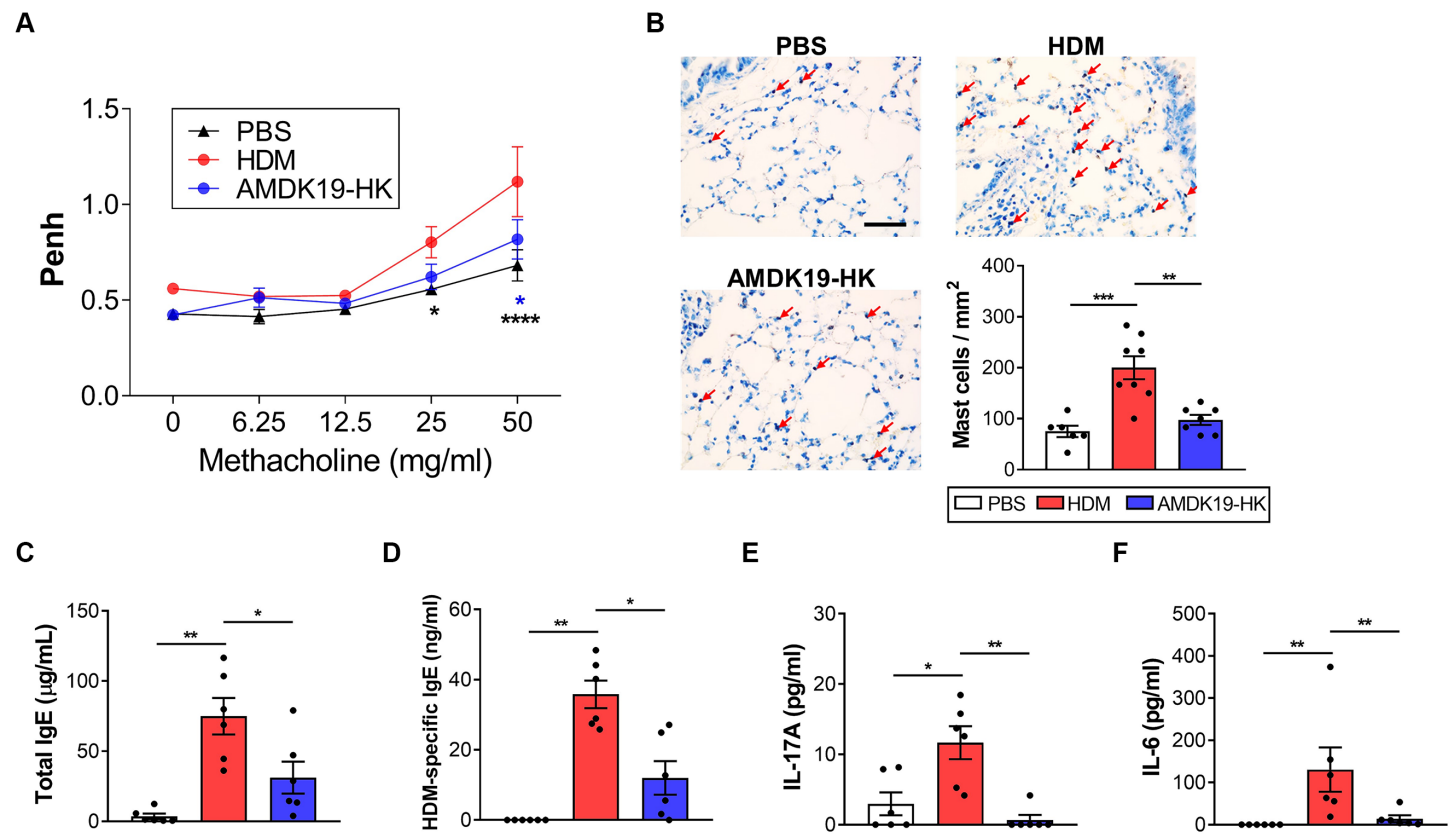
Effects of AMDK19-HK on airway hyperreactivity in HDM-induced AA model. **(A)** Changes in AHR in response to increasing doses of aerosolized methacholine were measured 24 h after the last HDM challenge with enhanced pause (Penh) parameter. **(B)** Toluidine blue staining of lung sections. Number of lung parenchymal mast cells were counted in high power fields (×400) per mm^2^. Scale bar, 100 μm. **(C,D)** Total IgE and HDM-specific IgE levels in serum. **(E,F)** The concentration of IL-17A and IL-6 (in pg./mL) in the BALF was quantified by ELISA. Data are expressed as mean ± SEM from a representative experiment of 3 performed (*n* = 6 mice per group). ns, not significant. ^*^p < 0.05, ^**^p < 0.01, ^****^p < 0.0001, two-way ANOVA with Bonferroni post-test **(A)** and Mann–Whitney *U*-test **(C–F)**. AHR, airway hyperresponsiveness.

Next, we evaluated the effect of AMDK19-HK on airway function by exposing the mice to increasing concentrations of methacholine. Compared to the HDM group, the AMDK19-HK group showed a significant decrease in AHR, as reflected by a decrease in Penh values, which were reversed by AMDK19-HK administration (Figure 2A). Because the accumulation and IgE-mediated activation of lung mast cells enhances methacholine-induced AHR (Stone et al., 2010), lung tissues were stained with toluidine blue to detect mast cells. Compared with the PBS group, a higher number of mast cells in the lung parenchymal tissues was observed in the HDM group, which was reversed by AMDK19-HK administration (Figure 2B). Furthermore, the total serum IgE and HDM-specific IgE levels were significantly higher in the HDM group than in the PBS group but were significantly lower in the AMDK19-HK group than in the HDM group (Figures 2C,D). As IL-17A produced by eosinophils in asthmatic lungs activates the release of IL-6 by bronchial fibroblasts, resulting in the enhancement of AHR (Molet et al., 2001), we further determined the concentrations of IL-17A and IL-6 in BALF by ELISA. The concentrations of IL-17A and IL-6 were significantly higher in the HDM group than in the PBS group but decreased in the AMDK19-HK group (Figures 2E,F). These results suggested that AMDK19-HK relieves HDM-induced AHR by decreasing IgE-mediated mast cell activation.

### 3.3 AMDK19-HK suppresses inflammatory leucocyte infiltration and Th2-associated cytokine and chemokine production in the BALF

**Figure 3:**
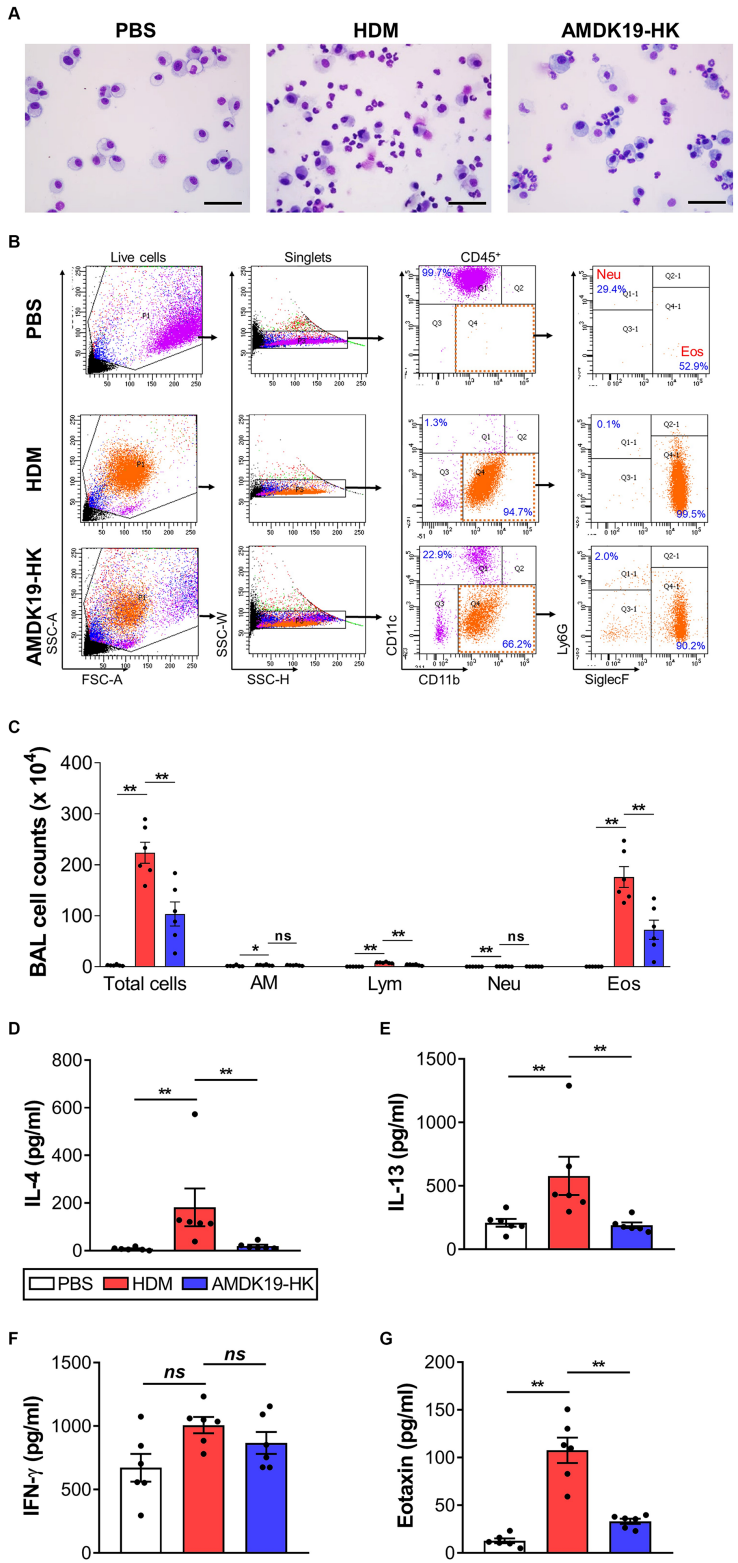
Effects of AMDK19-HK on leukocyte infiltration and cytokine secretion in BALF. Leukocytes were gated as live CD45^+^ cells. Percentage of eosinophils and neutrophils were calculated from the total number of live CD45^+^ cells. **(A)** Representative images of Diff-Quick-stained cytospins of BALF. Scale bar, 50 μm. **(B)** Gating strategy for the identification of BALF leukocytes. FSC-A and SSC-A were used to gate nucleated cells. SSC-W and SSC-H were used to gate singlets. Leukocytes were gated as live CD45^+^ cells. AMs were gated as CD45^+^CD11b^low^CD11c^+^ cells. Neutrophils were gated as CD45^+^CD11c^low^CD11b^+^Ly6G^+^Siglec-F^low^ cells. Eosinophils were gated as CD45^+^CD11c^low^CD11b^+^Ly6G^low^Siglec-F^+^ cells. **(C)** Count of total leukocytes, AM, Lym, Neu, and Eos in BALF. Each dot represents one mouse (*n* = 6 mice per group). **(D–G)** Cytokines levels (in pg/mL; IL-4, IL-13, and IFN-γ) and Eotaxin levels (in pg./mL) in BALF were quantified by ELISA. Each dot is the average of multiple measurements from one animal (*n* = 6 mice per group). Data are expressed as mean ± SEM from a representative experiment of 3 performed. ns, not significant. ^*^p < 0.05, ^**^p < 0.01, Mann–Whitney *U*-test. BALF, bronchoalveolar lavage fluid; CD, cluster of differentiation; SSC-A, side scatter area; FSC-A, forward scatter area; SSC-W, side scatter width; SSC-H, side scatter height; AM, alveolar macrophage; Lym, lymphocyte; Neu, neutrophil; Eos, eosinophil.

Since eosinophils are key to the induction of airway remodeling and AHR (Possa et al., 2013), we analyzed the number of inflammatory cells using cytospin and flow cytometry. Microscopic cytospin analysis revealed that total and differential leukocyte counts were substantially increased in the HDM group compared to those in the PBS group, but were decreased in the AMDK19-HK group (Figure 3A). Flow cytometry revealed a higher number of total and differential leukocytes, including macrophages, lymphocytes, neutrophils, and eosinophils, in the HDM group than in the PBS group (Figures 3B,C). Eosinophils constitute the majority of the CD45+ leukocytes. In contrast, the oral administration of AMDK19-HK led to a significant decrease in the number of all tested cell types, especially eosinophils.

Next, we assessed the effect of AMDK19-HK on cytokine and chemokine production in the BALF. The concentrations of Th2 cytokines IL-4, IL-5, IL-13, and a Th1 cytokine IFN-γ were measured by ELISA (Figures 3D–G). The concentrations of IL-4 and IL-13 were significantly higher in the HDM group than in the PBS group but were significantly lower in the AMDK19-HK group than in the HDM group (Figures 3D,E). IL-5 was detected at very low levels in the BALF of HDM mice (data not shown). Furthermore, a slight increase in IFN-γ levels was observed in the HDM group compared with the PBS group, albeit without statistical significance, but its levels were not altered by AMDK19-HK administration (Figure 3F). Moreover, we determined the concentration of the eosinophil-recruiting chemokine, eotaxin, using ELISA. As expected, AMDK19-HK significantly decreased HDM-induced eotaxin production (Figure 3G), suggesting that AMDK19-HK alleviates AAI by reducing the production of HDM-induced type 2 inflammatory mediators associated with eosinophil-driven inflammation.

### 3.4 AMDK19-HK reduces eosinophil infiltration and Th2-associated cytokine and chemokine expression in the lung tissues

**Figure 4:**
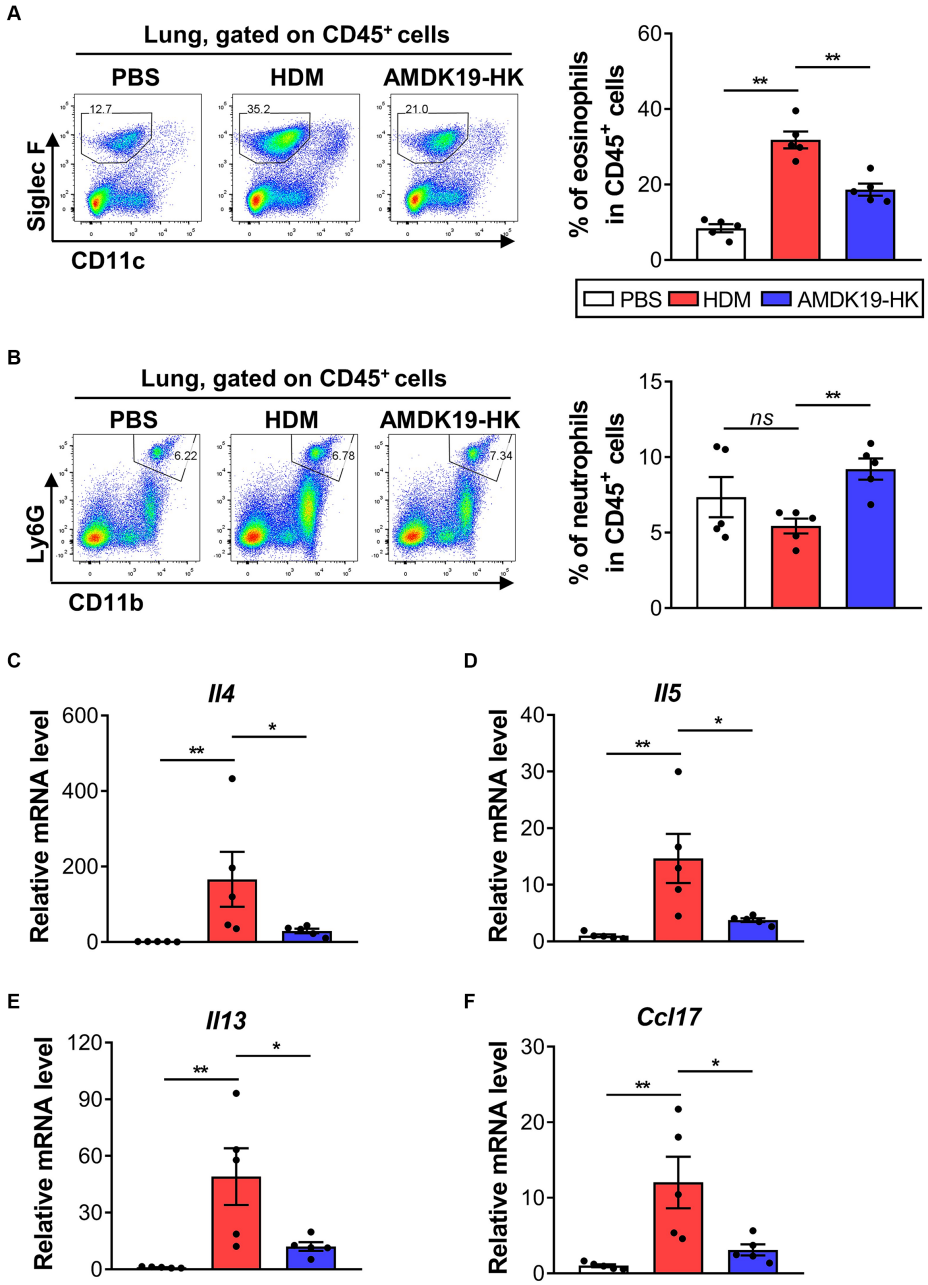
Effects of AMDK19-HK on eosinophil infiltration and cytokine/chemokine expression in lung tissues. **(A)** Gating strategy for lung eosinophils. Left: representative plots. Right: percentage of CD11c^low^Ly6G^low^Siglec-F^+^ eosinophils in the lung. Each dot represents one mouse (*n* = 5 mice per group). **(B)** Gating strategy for lung neutrophils. The representative plots (*left*) and the percentage of CD45^+^CD11b^+^Ly6G^high^ neutrophils in the lung (*right*). **(C–F)** The relative transcript levels of *Il4*, *Il5*, *Il13*, and *Ccl17* genes in lung tissues were measured by RT-qPCR. Expression levels were normalized to the housekeeping gene *Actb* and were expressed as a relative ratio. Each dot is the average of multiple measurements from one animal (*n* = 5 mice per group). Data are expressed as mean ± SEM from a representative experiment of 3 performed. ns, not significant. ^*^p < 0.05, ^**^p < 0.01, Mann–Whitney *U*-test. RT-qPCR, reverse transcription-quantitative real-time polymerase chain reaction.

To address the inflammatory responses in the lung tissues of the HDM and AMDK19-HK groups, we characterized the infiltration of eosinophils and neutrophils into the lung tissues using flow cytometry (Figures 4A,B). As expected, AMDK19-HK administration significantly reduced HDM-induced eosinophil infiltration but not neutrophil infiltration. We also assessed the expression of Th2 cytokines *Il4*, *Il5*, and *Il13* mRNAs and a potent Th2 cell chemoattractant *Ccl17* (*Tarc*) mRNA (Berin, 2002) in lung tissue homogenates using RT-qPCR (Figures 4A–F). Compared to the PBS group, the expression of all cytokine and chemokine genes analyzed in the HDM group was significantly elevated, which was significantly reduced by AMDK19-HK administration.

### 3.5 AMDK19-HK suppresses the production of Th2-associated cytokines in splenocytes on *in vitro* HDM restimulation

**Figure 5:**
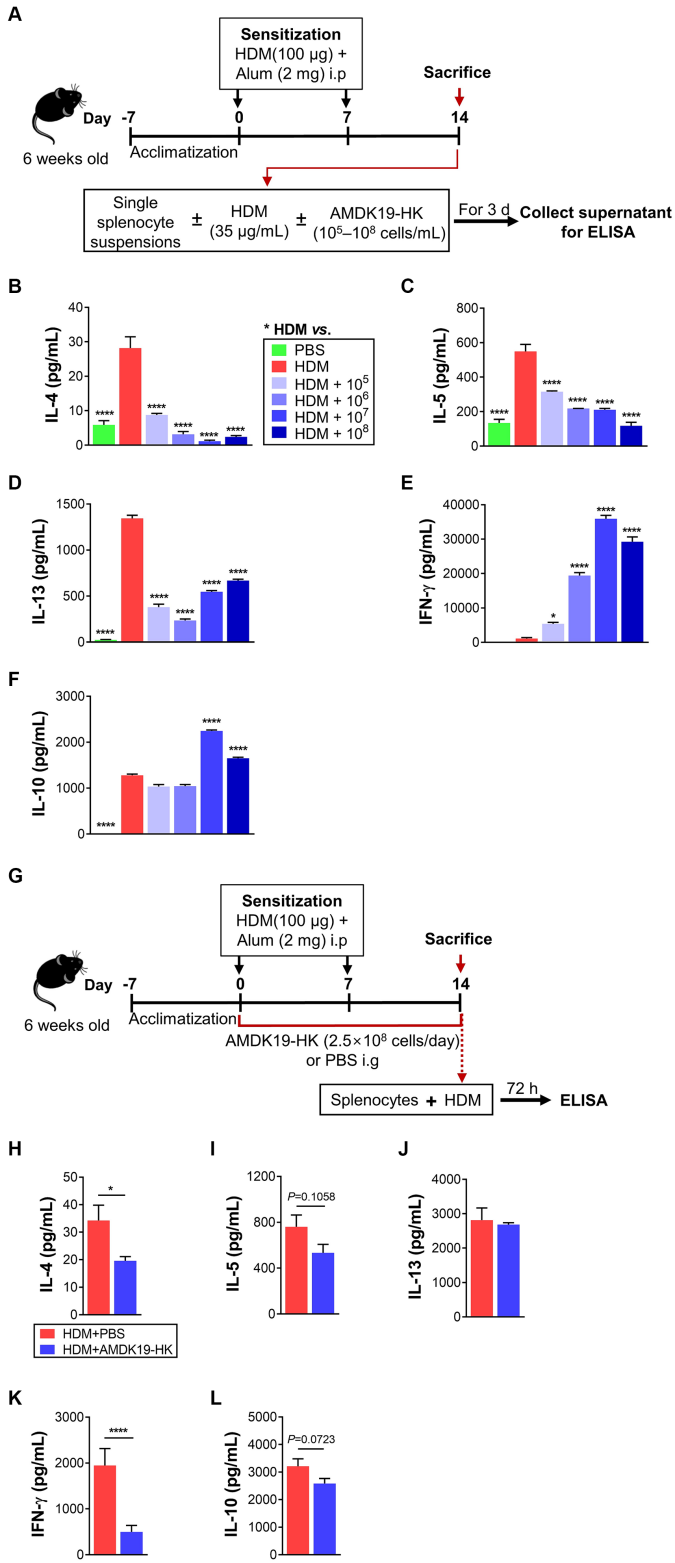
Effects of AMDK19-HK on cytokine production in splenocytes *in vitro* and *ex vivo*. **(A)** A schematic representation of the *in vitro* experiments. On days 0 and 7, mice were sensitized by intraperitoneal injection 100 μg of HDM/alum. On Day 14, splenocytes were isolated from HDM-sensitized mice and restimulated *in vitro* with HDM (35 μg/mL) in the presence or absence of AMDK19-HK at the indicated concentrations for 3 days. **(B–F)** The concentrations of IL-4, IL-5, IL-13, IL-10, and IFN-γ (in pg/mL) in the supernatants were quantified by ELISA. **(G)** A schematic representation of the *ex vivo* experiments. On days 0 and 7, mice were sensitized with HDM/alum. AMDK19-HK was given from 1 week before the first sensitization until the 1 day before the first challenge via oral gavage. On day 14, splenocytes were obtained and restimulated with HDM (35 μg/mL) for 3 days. **(H–L)** The concentrations of IL-4, IL-5, IL-13, IL-10, and IFN-γ (in pg/mL) in the supernatants. Data are expressed as mean ± SEM from a representative experiment of 3 performed. ^*^p < 0.05 and ^****^p < 0.0001 versus HDM group, one-way ANOVA with Tukey post-test **(B–F)** and unpaired two-tailed Student’s *t*-test **(H–L)**.

To further investigate the immunomodulatory effects of AMDK19-HK on T-cell responses to HDM, splenocytes (5 × 10^6^ cells/mL) obtained from mice were sensitized by two intraperitoneal (i.p.) injections with HDM and incubated with PBS or HDM (35 μg/mL) with or without increasing doses of AMDK19-HK (1 × 10^5^, 1 × 10^6^, 1 × 10^7^ and 1 × 10^8^ cells/mL) for 3 days (Figure 5A). The concentrations of cytokines (IL-4, IL-5, IL-13, IL-10, and IFN-γ) in the supernatant were measured by ELISA. Compared with PBS control, HDM restimulation significantly increased IL-4, IL-5, and IL-13 levels, but not IFN-γ levels (Figures 5B–E), which is indicative of HDM-specific Th2 cell responses. In contrast, HDM restimulation with AMDK19-HK dose-dependently decreased IL-4 and IL-5 levels and significantly suppressed IL-13 levels at all examined concentrations (Figures 5B–D). Moreover, IFN-γ levels were markedly elevated in a dose-dependent manner, and IL-10 levels were significantly increased only at two high-doses of AMDK19-HK (1 × 10^7^ and 1 × 10^8^ cells; Figures 5E,F). These observations indicated that AMDK19-HK alleviated Th2-mediated airway inflammation by rebalancing the Th1/Th2 immune response and modulating the IL-10 anti-inflammatory immune response.

We further performed an *ex vivo* experiment with splenocytes obtained from HDM-sensitized mice administered daily with either PBS (vehicle) or AMDK19-HK (2.5 × 10^8^ cells/day) during the experimental period (Figure 5G). Compared with splenocytes from PBS-treated HDM-sensitized mice, HDM restimulation of those from AMDK19-HK-treated HDM-sensitized mice significantly decreased IL-4 and IFN-γ levels (Figures 5H,K), and moderately reduced IL-5 and IL-10 levels (*p* = 0.1058 and *p* = 0.0723, respectively; Figures 5I,L); however, IL-13 levels were unchanged (Figure 5J). These results suggest that oral administration of AMDK19-HK suppresses T cell responsiveness to HDM in the systemic immune system, thereby ameliorating AAI in a mouse model of AA.

### 3.6 AMDK19-HK alters the relative abundance of specific SCFA-associated cecal microbiota

**Figure 6:**
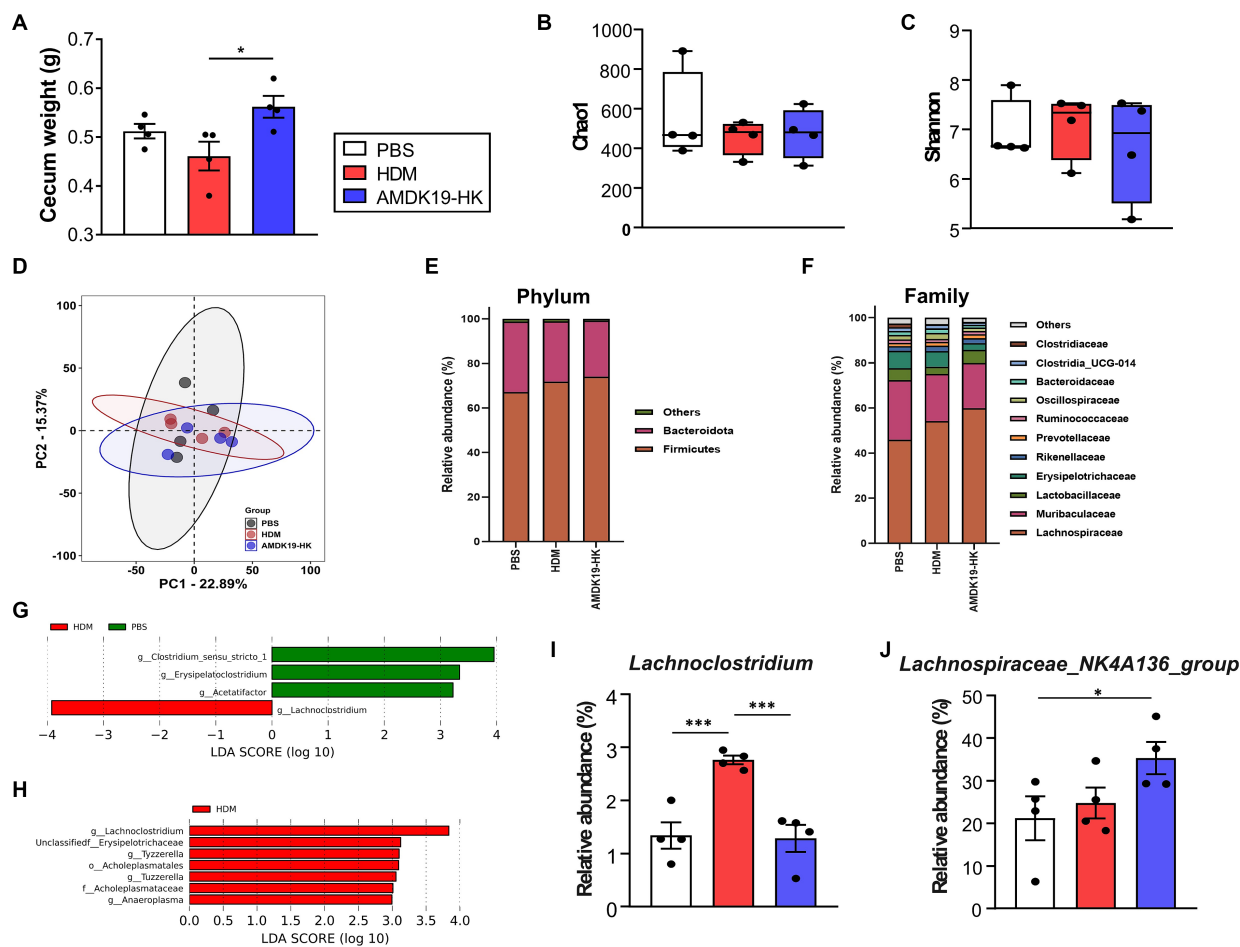
Characteristics of cecal microbiota in experimental groups subjected to PBS, HDM, and AMDK19-HK. **(A)** Total cecum weight (*n* = 4 per group, ^*^p < 0.05, Mann–Whitney *U*-test). **(B,C)** The α diversity indices measured by **(B)** Chao1 and **(C)** Simpson methods. The boxplots show IQR ranges with the median and whiskers extending to the most extreme point within 1.5 times the IQR. **(D)** Comparison of the β diversity of the intestinal microbiota using PCoA based on the Bray–Curtis dissimilarity matrix. **(E)** Average relative abundance of prevalent microbiota at the phylum level. **(F)** Average relative abundance of the top 22 bacteria at the family level. **(G)** LEfSe analysis between the PBS and HDM groups (LDA [log10] score > 2 and *p* < 0.05). **(H)** LEfSe analysis between the HDM and AMDK19-HK groups. **(I)** The relative abundance of *Lachnoclostridum* in cecal samples in all three groups. **(J)** The relative abundance of *Lachnospiraceae_NK4A136 group* in cecal samples in all three groups. ^*^p < 0.05, ^***^p < 0.001. IQR, interquartile; PCoA, principal coordinate analysis; LEfSe, linear discriminant analysis (LDA) effect size.

Here, we found that cecum weight was significantly higher in the AMDK19-HK group than in the HDM group (Figure 6A). As changes in cecum size can reflect changes in gut microbial composition (Fujisaka et al., 2018), we investigated the impact of AMDK19-HK on cecal bacterial community structure in an HDM-induced AA mouse model. Cecal DNA from the PBS, HDM, and AMDK19-HK groups was sequenced to identify the hypervariable V1-V2 region of the 16S rRNA gene. The Chao1 and Simpson alpha diversity indices exhibited slight fluctuations, but the differences in microbial richness and diversity between the groups did not reach statistical significance (Kruskal–Wallis, *p* > 0.05; Figures 6B,C). Beta diversity (measured by Bray–Curtis dissimilarity) was also not significantly different among the cecal bacterial communities in the three groups (PERMANOVA, *p* > 0.05; Figure 6D). Thus, we focused on the specific effects of AMDK19-HK on the microbial community abundance and composition. At the phylum level, the two most dominant phyla were *Firmicutes* and *Bacteroidota* in the three groups (Figure 6E). Compared with the PBS group, the relative abundance of *Firmicutes* showed an increasing trend (PBS, 67.13 ± 6.28% vs. HDM, 71.83 ± 5.00%, and PBS, 67.13 ± 6.28% vs. AMDK19-HK, 74.10 ± 3.21%, respectively; Supplementary Table S3), while the relative abundance of *Bacteroidota* showed a decreasing trend in the HDM and AMDK19-HK groups (PBS, 31.68 ± 6.06% vs. HDM, 27.03 ± 4.81%, and PBS, 31.68 ± 6.06% vs. AMDK19-HK, 25.16 ± 3.06%, respectively); however, there was no statistical difference between the HDM and AMDK19-HK groups. At the family level, the two most dominant families were *Lachnospiraceae* and *Muribaculaceae* (Figure 6F). Notably, the relative abundance of *Lachnospiraceae* was higher in the AMDK-19-HK group than in the PBS and HDM groups, but the differences between the HDM and AMDK19-HK groups did not reach statistical significance (PBS, 45.98 ± 14.83% vs. HDM, 54.14 ± 3.76%, and PBS, 45.98 ± 14.83% vs. AMDK19-HK, 59.95 ± 4.94%, respectively; Supplementary Table S3).

Using LEfSe analysis (logarithmic LDA score >2.0, *p* < 0.05), differences in the cecal microbiota composition were identified (Figures 6G,H). The genera *Clostridium_sensu_stricto_1 group*, *Acetatifactor*, and *Erysipelatoclostridium* were enriched in the PBS group, whereas *Lachnoclostridium* was enriched in the HDM group (Figure 6G; Supplementary Table S4). The genera *Lachnoclostridium*, *Erysipelotrichaceae*, *Tuzzerella*, *Anaeroplasma*, and *Tyzzerella* were enriched in the HDM group, whereas none were enriched in the AMDK19-HK group (Figure 6H; Supplementary Table S5).

The relative abundance of the genus *Lachnoclostridium* which is negatively correlated with circulating acetate levels (Nogal et al., 2021), was considerably higher in the HDM group than in the PBS group (*p* < 0.001; Figure 6I), whereas AMDK19-HK administration significantly decreased its abundance to levels equivalent to those of the PBS controls in the HDM group (*p* < 0.001, HDM vs. AMDK19-HK). Furthermore, the relative abundance of the main SCFA-producing *Lachnospiraceae_NK4A136_group* (Li et al., 2020) was higher in the AMDK19-HK group than that in the PBS and HDM groups (Figure 6J; Supplementary Table S3).

Collectively, these results suggest that the oral administration of AMDK19-HK modulates the relative abundance of specific SCFA-associated cecal genera in an HDM-induced AA mouse model.

### 3.7 AMDK19-HK enhances cecal SCFA production and intestinal mucosal barrier function

**Figure 7:**
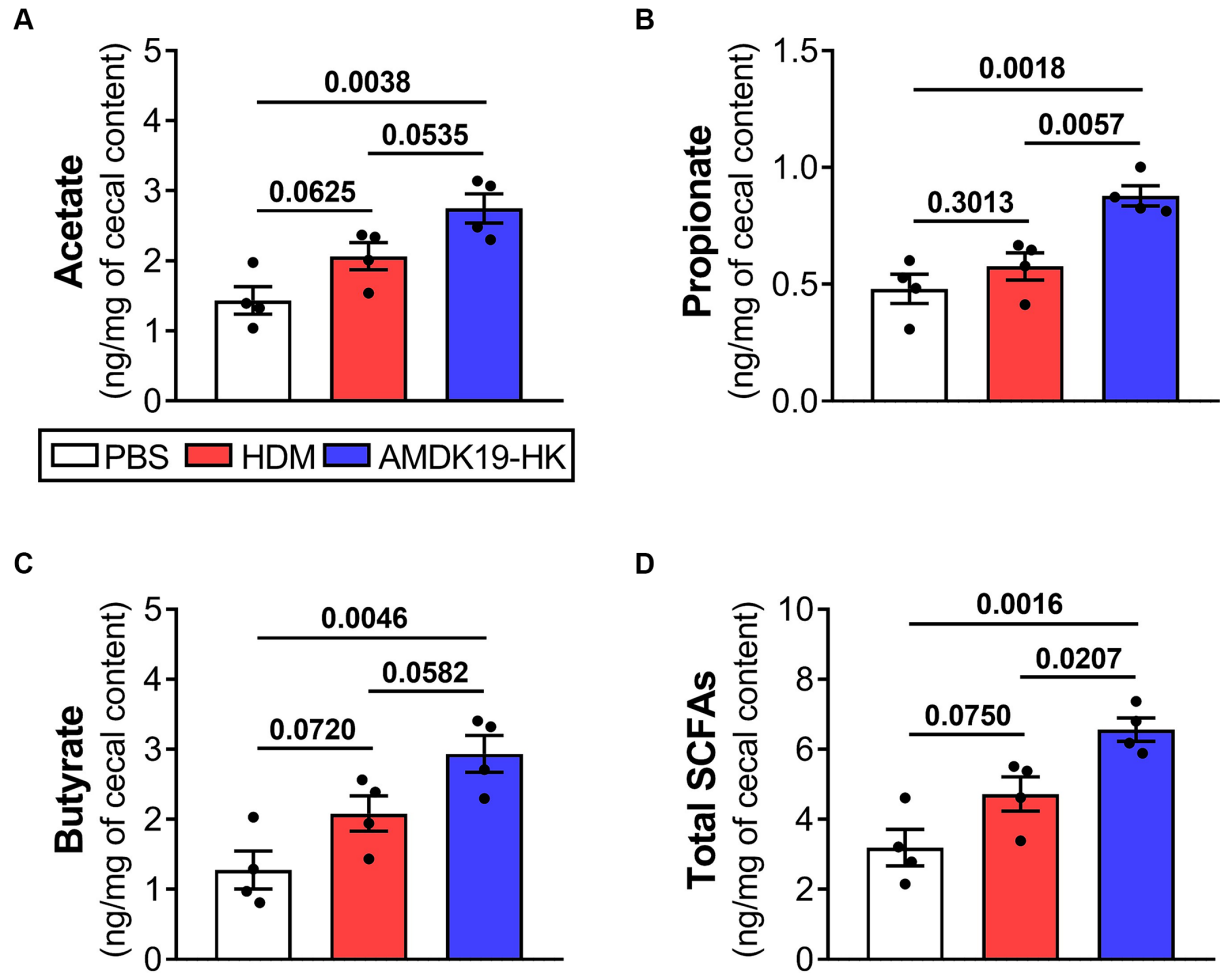
Effects of AMDK19-HK administration on the production of cecal SCFAs in HDM-induced AA model. GC–MS quantification of SCFA acetate **(A)**, propionate **(B)**, butyrate **(C)**, and total SCFAs [sum of acetate, propionate, and butyrate; **(D)**] in cecal contents taken from the PBS, HDM, and AMDK19-HK groups (*n* = 4 per group). SCFA concentrations are expressed per mg of cecal content wet weight. Data are expressed as mean ± SEM and statistical significance was determined by Student’s *t*-test. GC–MS, gas chromatography–mass spectrometry.

Gut microbiota-generated SCFAs, including acetate, propionate, and butyrate, are abundant in the cecum and ascending colon (Cummings et al., 1987) and circulating SCFAs can influence AA-related immune functions (Verstegen et al., 2021). Thus, we conducted a targeted metabolomic analysis to measure the concentrations of acetate, propionate, butyrate, and total SCFAs (sum of acetate, propionate, and butyrate) in cecal contents (Figure 7). Concentrations of individual and total SCFAs did not differ between the PBS and HDM groups. However, compared with the PBS group, the concentrations of acetate, propionate, butyrate, and total SCFAs in the AMDK19-HK group were notably elevated (*p* = 0.0038, *p* = 0.0018, *p* = 0.0046, and *p* = 0.0016, respectively; Figures 7A–D). Furthermore, compared to the HDM group, the AMDK19-HK group showed significant increases in the levels of propionate (*p* = 0.0057; Figure 7B) and total SCFAs (*p* = 0.0207; Figure 7D), and an upward trend in acetate (*p* = 0.0535; Figure 7A) and butyrate levels (*p* = 0.0582; Figure 7C).

**Figure 8:**
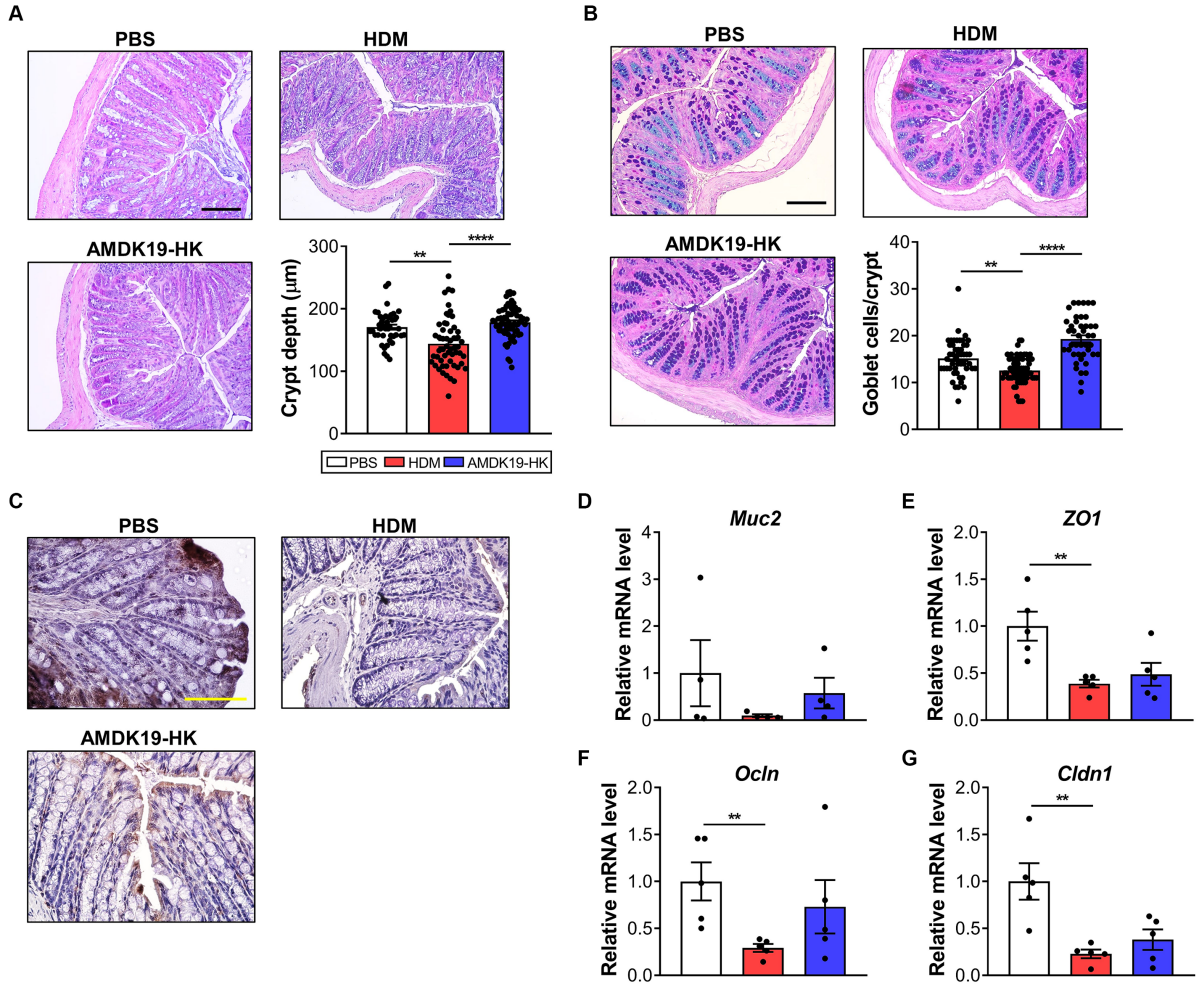
Effect of AMDK19-HK on the intestinal mucosal barrier function. **(A)** Representative image of H&E-stained colonic sections of each group (*left*) and crypt depth of colon (*right,* n = 20 crypts in 3 mice). Scale bar, 100 μm. **(B)** Representative images of AB-PAS-stained colonic sections of mice (*left*) and goblet cell counts per crypt (*right*). Scale bar, 100 μm. **(C)** Representative images of Zo-1-stained colonic sections of mice. Scale bar, 10 μm. **(D–G)** The relative transcript levels of *Muc2*, *Zo-1*, *Ocln*, and *Cldn1* genes in the colon were measured by RT-qPCR. Expression levels were normalized to the housekeeping gene *Gapdh* and were expressed as a relative ratio. Data are expressed as mean ± SEM from a representative experiment of 3 performed (*n* = 5 mice per group). ns, not significant. ^*^p < 0.05, ^**^p < 0.01, ^****^p < 0.0001, Mann–Whitney *U*-test. AB-PAS, alcian blue-periodic acid-Schiff; *Muc2*, mucin 2; *Zo1*, zonula occludens-1; *Ocln*, occludin; *Cldn1*, claudin-1.

Because SCFAs are responsible for intestinal epithelial protection by increasing mucus synthesis and tight junction protein-encoding gene expression (Iacob and Iacob, 2019), we assessed the effect of AMDK19-HK on the morphology and expression of intestinal barrier-related genes in the colons of the three groups (Figure 8). Histopathological analysis revealed that crypt depth and mucus-producing goblet cell numbers were significantly higher in the AMDK19-HK group than in the HDM group (Figures 8A–C). Consistently, the mRNA expression of Mucin 2 (*Muc2*) increased in the AMDK19-HK group (Figure 8D). Furthermore, the expression levels of tight junction protein-encoding genes, including Zonula occludens-1 (*ZO1*), Occludin (*Ocln*), and Claudin-1 (*Cldn1*), were higher in the AMDK19-HK group than in the HDM group, although the difference was not statistically significant (Figures 8E–G).

Taken together, these results suggest that the suppressive effect of AMDK19-HK on AAI in the mouse model of AA may be associated with increased cecal SFCAs levels and enhanced gut barrier function.

## 4 Discussion

The high prevalence, heterogeneity, and incurability of asthma necessitate the development of novel therapies. In a previous study, mice orally administered the live form of *A. muciniphila* exhibited reduced AAI (Michalovich et al., 2019), suggesting its potential therapeutic utility. Herein, we demonstrated the mechanism of action of *A. muciniphila* and the potential effects of its heat-killed form on AAI. Oral administration of AMDK19-HK, killed by pasteurization, mitigated AAI, airway remodeling, AHR to inhaled methacholine, mast cell accumulation, serum HDM-specific IgE levels, and eosinophil accumulation in mice with HDM-induced AA. AMDK19-HK decreased Th2-associated cytokine and chemokine production in the lung tissue and BALF. Furthermore, AMDK19-HK suppressed Th2-associated cytokine levels after *in vitro* HDM restimulation in splenocytes of HDM-sensitized mice. Additionally, AMDK19-HK modulated the relative abundance of specific SCFA-associated cecal genera and cecal levels of SCFAs, and enhanced intestinal mucosal barrier function in a mouse model of AA.

AA pathogenesis involves cytokine-mediated crosstalk among airway epithelial, smooth muscle, and immune cells, leading to airway inflammation, airway remodeling, and AHR (Kubo, 2017; Caminati et al., 2018; Yang et al., 2021). We demonstrated that AMDK19-HK administration mitigated HDM-induced airway eosinophilia, quantifiable in BALF and lung tissues, and structural changes, including goblet cell hyperplasia and subepithelial collagen deposition in lung tissues. Specifically, eosinophil recruitment to the lungs is crucial in AA pathogenesis, releasing multiple inflammatory mediators, such as eosinophil cationic protein, eosinophil peroxidase, major basic protein, and eosinophil-derived neurotoxin, resulting in the induction of airway epithelial cell damage, mucus hypersecretion, and collagen deposition (Possa et al., 2013). It is additionally noteworthy that AMDK19-HK was slightly more efficient than live AMDK19 in alleviating HDM-induced peribronchial cellular recruitment, goblet cell hyperplasia, and eosinophilia in the airways (Supplementary Figure S2). We also observed that AMDK19-HK administration reduced AHR to methacholine inhalation, lung mast cell accumulation, and serum HDM-specific IgE levels. Furthermore, AMDK19-HK considerably downregulated HDM-induced expression of IL-6 and IL-17A. IgE is key in the initiation and maintenance of airway inflammation (Méndez-Enríquez and Hallgren, 2019). Upon allergen challenge, mast cells are activated by IgE-mediated crosslinking of high-affinity IgE receptors (FcεRI) with allergens, releasing inflammatory mediators with airway-constrictive effects, including histamine, protease, leukotriene, and prostaglandins (Méndez-Enríquez and Hallgren, 2019). Moreover, IgE can promote FcεRI-mediated airway smooth muscle (ASM) cell proliferation and contraction, and the release of cytokines and chemokines in ASM cells, which contribute to AHR and asthma severity (Méndez-Enríquez and Hallgren, 2019). Additionally, IL-17A from eosinophils in asthmatic lungs triggers IL-6 release by bronchial fibroblasts in asthmatic airways, enhancing extracellular matrix deposition and AHR (Molet et al., 2001). Collectively, our data suggest that AMDK19-HK administration attenuates the pathophysiological severity of airway remodeling and AHR by suppressing IgE-mediated activation of mast and ASM cells in an HDM-induced AA mouse model.

AMDK19-HK administration significantly decreased the production of IL-4, IL-5, IL-13, eotaxin, and CCL17 (TARC), but not IFN-γ, in the lungs. Th2 cells and their cytokines, IL-4, IL-5, and IL-13, are considered the main orchestrators of AAI, airway remodeling, and AHR, in both humans and mice (Bošnjak et al., 2019; Akdis et al., 2020). IL-4 promotes B cell IgE isotype switching and IgE synthesis, IL-13 increases mucus hypersecretion and eotaxin production, and IL-5 promotes the maturation, survival, and recruitment of eosinophils (Akdis et al., 2020). Furthermore, the expression of the Th2-attracting chemokine TARC in DCs is reportedly upregulated in the airways of patients with AA following allergen challenge, thereby promoting the recruitment of Th2 cells and eosinophils into the lungs during AA development (Berin, 2002). Therefore, AMDK19-HK administration may restore the Th1/Th2 balance to specifically suppress Th2 cytokine-mediated responses, thereby alleviating AAI in a mouse model of AA.

Systemic allergen sensitization generates circulating CD4^+^ Th cells that migrate from the spleen to the lungs, where they amplify the allergen-specific effector CD4^+^ T cell population to initiate AA relapse (Bošnjak et al., 2019). The *in vitro* restimulation experiment with splenocytes from HDM-sensitized mice revealed that HDM stimulation significantly increased IL-4, IL-5, IL-10, and IL-13 production, but not IFN-γ production; however, HDM plus AMDK19-HK stimulation significantly downregulated IL-4, IL-5, and IL-13 production, but upregulated IFN-γ and IL-10 production. Therefore, AMDK19-HK suppresses Th2-mediated immune responses to HDM by rebalancing the Th1/Th2 immune response and inducing an anti-inflammatory immune response. Furthermore, an *ex vivo* HDM restimulation experiment with splenocytes obtained from HDM-sensitized mice administered AMDK19-HK daily revealed decreased Th1 and 2 responses, along with decreased IL-10 production. Additionally, HDM-challenged mice orally administered AMDK19-HK exhibited higher splenic Treg frequency (Supplementary Figure S3). Oral administration of the probiotic *Lactiplantibacillus plantarum*, despite the absence of colonization, can modulate intestinal and systemic immune responses by sensing probiotics using specific DCs in the intestinal lamina propria or Peyer’s patches (Bermudez-Brito et al., 2018). These regulatory CD103^+^ DCs migrate to the spleen, leading to increased Treg cell frequencies and decreased splenic Th-cell cytokine responses (Bermudez-Brito et al., 2018). Collectively, AMDK19-HK administration enhances Treg frequency and suppresses T-cell responsiveness in systemic circulation, thereby alleviating AAI in a mouse model of AA. Further studies should elucidate how DC-driven Treg cell generations by AMDK19-HK helps control AAI.

Cecal *16S rRNA* sequence analysis revealed that AMDK19-HK administration faintly altered the gut microbial community structure. Nevertheless, AMDK19-HK administration to an HDM-induced AA mouse model significantly increased the number of the main SCFA-producing *Lachnospiraceae_NK4A136_group* (Li et al., 2020). Gut microbiota-generated SCFAs are abundant in the cecum and ascending colon, and influence host immunity and disease risk both in the intestine and the periphery (Cummings et al., 1987). Intestinal SCFAs migrate into pulmonary circulation and affect AA-related immune functions (Verstegen et al., 2021). Notably, the relative abundance of the *Lachnospiraceae_NK4A136_group* is negatively correlated with inflammatory serum markers in ovalbumin-induced AA mice (Liao et al., 2022). We also observed significantly decreased *Lachnoclostridium* numbers in this study. The relative abundance of *Lachnoclostridium* is positively correlated with eosinophilic inflammation in chronic rhinosinusitis with nasal polyps (Kim et al., 2020). Furthermore, *Lachnoclostridium* negatively influences circulating acetate levels and produces trimethylamine (TMA), which is rapidly absorbed into the circulation and converted to potentially harmful trimethylamine-N-oxide (TMAO) in the liver (Nogal et al., 2021). This increases the risk of cardiometabolic diseases, including obesity, metabolic syndrome, and atherosclerosis (Nogal et al., 2021). Elevated circulating TMAO levels promote systemic inflammation and oxidative stress (El Hage et al., 2023). The positive association between atherosclerosis and AA (Liu et al., 2016) and the additive effects of obesity on asthma risk and severity are noteworthy (Michalovich et al., 2019). Therefore, the *Lachnospiraceae_NK4A136_group* may play an important role in the prevention of AAI by producing SCFAs, while *Lachnoclostridium* may have a negative effect on AA by influencing circulating acetate levels and producing harmful metabolites, such as TMA. Concordantly, AMDK19-HK administration significantly increased cecal SCFAs levels (acetate, propionate, and butyrate). Moreover, Spearman’s correlation analysis showed that the abundance of the *Lachnospiraceae_NK4A136_group* was positively correlated with cecal propionate concentration (Spearman’s rho = 0.69, *p* = 0.0173; Supplementary Table S6). SCFAs exert their effects by activating G-protein-coupled receptors (GPCRs) and inhibiting histone deacetylases (Rooks and Garrett, 2016). Notably, GPCRs (GPR41, GPR43, and GPR109A) are expressed in immune cells, including eosinophils, B cells, T cells, DCs, and mast cells, and are involved in the sensitization and effector phases of AA (Kubo, 2017; Verstegen et al., 2021). Additionally, a previous study using a mouse model of AA demonstrated that asthma pathogenesis is closely associated with histone acetylation modifications in airway epithelial and ASM cells, eosinophils, and perivascular inflammatory cells in asthmatic lung tissues (Ren et al., 2021). Given that (i) acetate, propionate, and butyrate reduce IgE isotype switching and eosinophil infiltration into the lung, (ii) propionate and butyrate inhibit FcεR-related gene expression in lung mast cells, (iii) propionate decreases IL-13 and airway mucus production, and (iv) butyrate upregulates the tolerogenic responses of DCs in the intestine (Martin-Gallausiaux et al., 2021; Verstegen et al., 2021), AMDK19-HK-induced increases in cecal SCFAs may skew immune response toward a local and systemic anti-inflammatory phenotype.

Increased cecal concentrations of SCFAs in the AMDK19-HK group were accompanied by enhanced levels of intestinal barrier function-related markers, including crypt depth, goblet cell count, and tight junction protein-encoding genes (*ZO1*, *Ocln*, and *Cldn1*). SCFAs can play crucial roles in promoting goblet cell differentiation, mucus production, and tight junction protein expression in colon crypts, which in turn stabilizes epithelial integrity and improves intestinal barrier function (Rooks and Garrett, 2016; Martin-Gallausiaux et al., 2021). Notably, intestinal barrier dysfunction contributes to the onset of symptoms in patients with HDM AA (Baioumy et al., 2021). Moreover, the relative abundance of the *Lachnospiraceae_NK4A136_group* is negatively correlated with intestinal barrier dysfunction and inflammation in a mouse model of dextran sodium sulfate-induced colitis (Li et al., 2020). Therefore, our data suggest that AMDK19-HK-induced increases in cecal SCFA concentrations could contribute to improved gut barrier function in HDM-induced AA mice. Currently, the mechanisms by which AMDK19-HK affects gut microbiota and SCFAs remain unclear. Moreover, we could not measure plasma SCFA levels because only a small amount of colon-derived SCFAs entered systemic circulation (Cummings et al., 1987). Furthermore, an association study between SCFAs and the gut microbiota was performed using 16S rRNA gene sequencing data, which are limited to identifying bacteria at the species or strain level and the metabolic pathways encoded by the microbiome. Thus, we plan to perform shotgun metagenomic sequencing analyses and investigate the potential roles of SCFAs and TMA in AA in the future. Additionally, Amuc_1100, a highly abundant outer membrane protein of *A. muciniphila*, could contribute to improving intestinal barrier function in HDM-induced AA mice because it is thermostable during pasteurization and has been demonstrated to ameliorate intestinal epithelial barrier damage by activating Toll-like receptor 2 (TLR2) and upregulating tight junction proteins in HFD-induced obese mice (Plovier et al., 2017).

Conclusively, our findings demonstrate that oral AMDK19-HK administration exerts a protective effect against HDM-induced AAI in mice by suppressing local and systemic Th2-mediated immune responses to HDM, modulating the relative abundance of specific SCFA-associated cecal microbiota and cecal levels of SCFAs, and enhancing intestinal mucosal barrier function. Our observations highlight the health benefits of AMDK19-HK, a potentially safe and feasible strategy for the prevention and amelioration of AA.

## Data availability statement

The datasets presented in this study can be found in online repositories. The names of the repository/repositories and accession number(s) can be found at: https://dataview.ncbi.nlm.nih.gov/object/PRJNA1074002?reviewer=v4mgq4b7jjoacolurefn8geisu, PRJNA1074002.

## Ethics statement

The animal study was approved by the Ethics Committee and Institutional Animal Care and Use Committee of Dongguk University Health System (IACUC-2022-029-1). The study was conducted in accordance with the local legislation and institutional requirements.

## Author contributions

SY: Methodology, Writing – original draft, Data curation, Formal analysis, Project administration, Visualization. YL: Data curation, Formal analysis, Methodology, Visualization, Writing – original draft. HB: Validation, Writing – original draft, Formal analysis, Data curation. JJ: Data curation, Formal analysis, Validation, Writing – original draft. SM: Data curation, Formal analysis, Validation, Writing – original draft, Visualization. M-GH: Data curation, Formal analysis, Visualization, Writing – original draft, Software. DK: Methodology, Writing – original draft, Formal analysis. E-JS: Formal analysis, Writing – original draft, Data curation, Software, Visualization. Y-DN: Writing – original draft, Methodology, Conceptualization, Investigation, Funding acquisition. J-GS: Conceptualization, Investigation, Writing – review & editing, Resources. S-NL: Conceptualization, Investigation, Resources, Writing – review & editing, Methodology, Supervision, Writing – original draft.

## Footnotes

^1^https://dataview.ncbi.nlm.nih.gov/object/PRJNA1074002?reviewer=v4mgq4b7jjoacolurefn8geisu
