# Supplementary material for: Heat-killed Akkermansia muciniphila ameliorates allergic airway inflammation in mice
Source: Front Microbiol. 2024 May 9;15:1386428. doi: 10.3389/fmicb.2024.1386428 (PMC11111871; doi:10.3389/fmicb.2024.1386428)
Supplement: Supplementary file 1 [file Data_Sheet_1.PDF]

Supplementary Material

1 Supplementary Data

1.1 Supplementary Figures

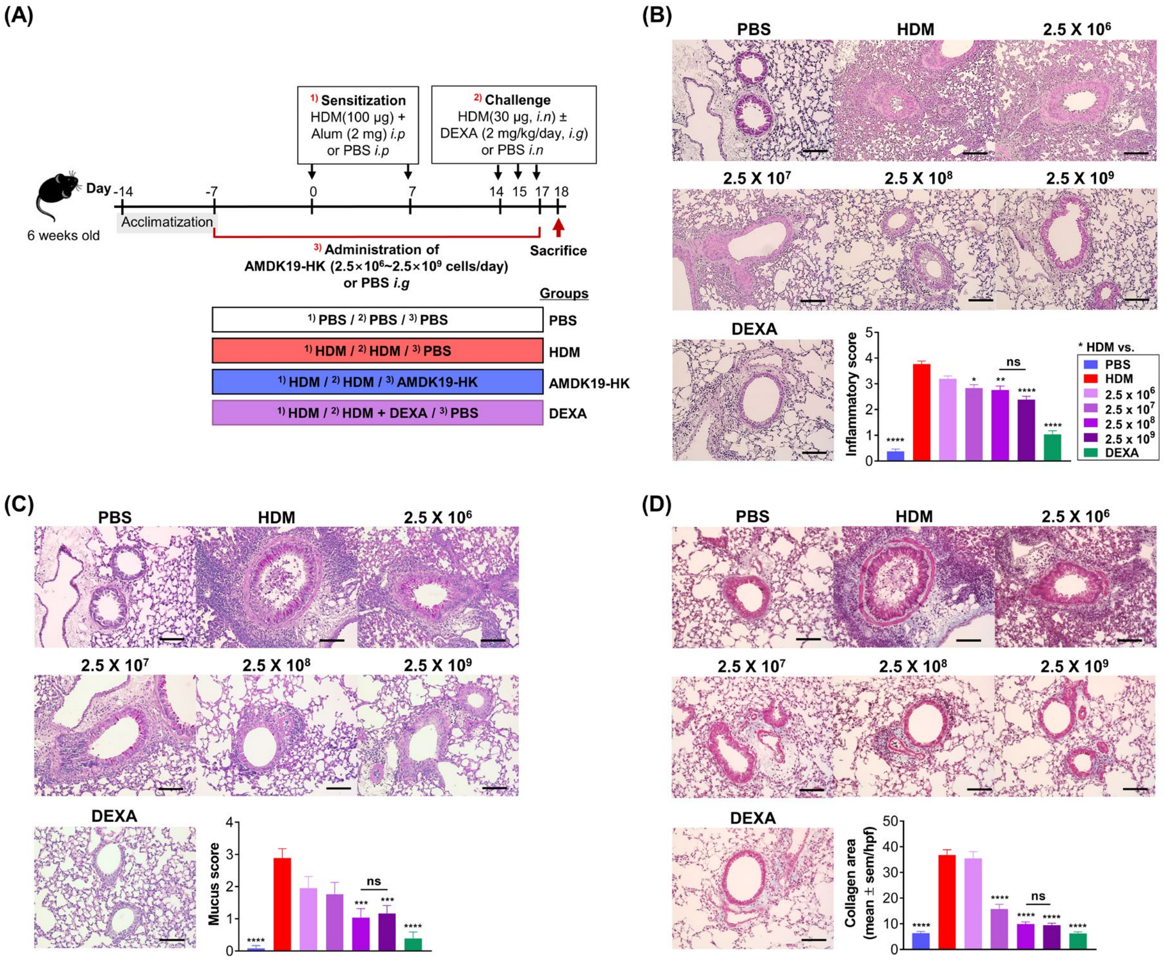

Supplementary Figure S1. Oral administration of AMDK19-HK inhibited HDM-induced airway remodeling. (A) Experimental design of the AA study on mice. A mouse model of AA mice was established by sensitization with HDM extracts and consequent challenge with HDM. AMDK19-HK was prepared by pasteurization for 30 min at 70°C. The AMDK19-HK group were given AMDK19-HK at increasing doses from ranging 2.5×10^6 to 2.5×10^9 cells/day via oral gavage from one week before the first sensitization until the end of the experiment. Mice treated with PBS under the same experimental condition were used as an untreated control. DEXA (2 mg/kg body weight/day) was used as positive control for treatment. On the end day of the experiment, lungs were collected, fixed, embedded in paraffin, and sectioned at 4-µm thickness. A representative of each group is shown (n = 5 mice/group). (B) H&E-stained slides of each

group. Scale bar, 100 μm. For semi-quantification, inflammatory cell infiltration was scored based on 5-point scale (0-4). (C) PAS-stained slides of each group. For semi-quantification, distribution of goblet cells and mucus production were scored based on 5-point scale (0-4). (D) M-T-stained slides of each group. Blue regions indicate collagen deposition. Semi-quantification of collagen deposition was performed by ImageJ software. Data are expressed as mean ± SEM for n=5-6 mice per group. * $P < 0.05$, ** $P < 0.01$, *** $P < 0.001$, and **** $P < 0.0001$, by nonparametric Kruskal–Wallis test with Dunn's posttest. i.p, intraperitoneal sensitization; i.n, intranasal challenge; i.g, intragastric injection; AA, allergic asthma; DEXA, dexamethasone; H&E, hematoxylin and eosin; HDM, house dust mite; PAS, Periodic acid- Schiff; M-T, Masson's trichrome.

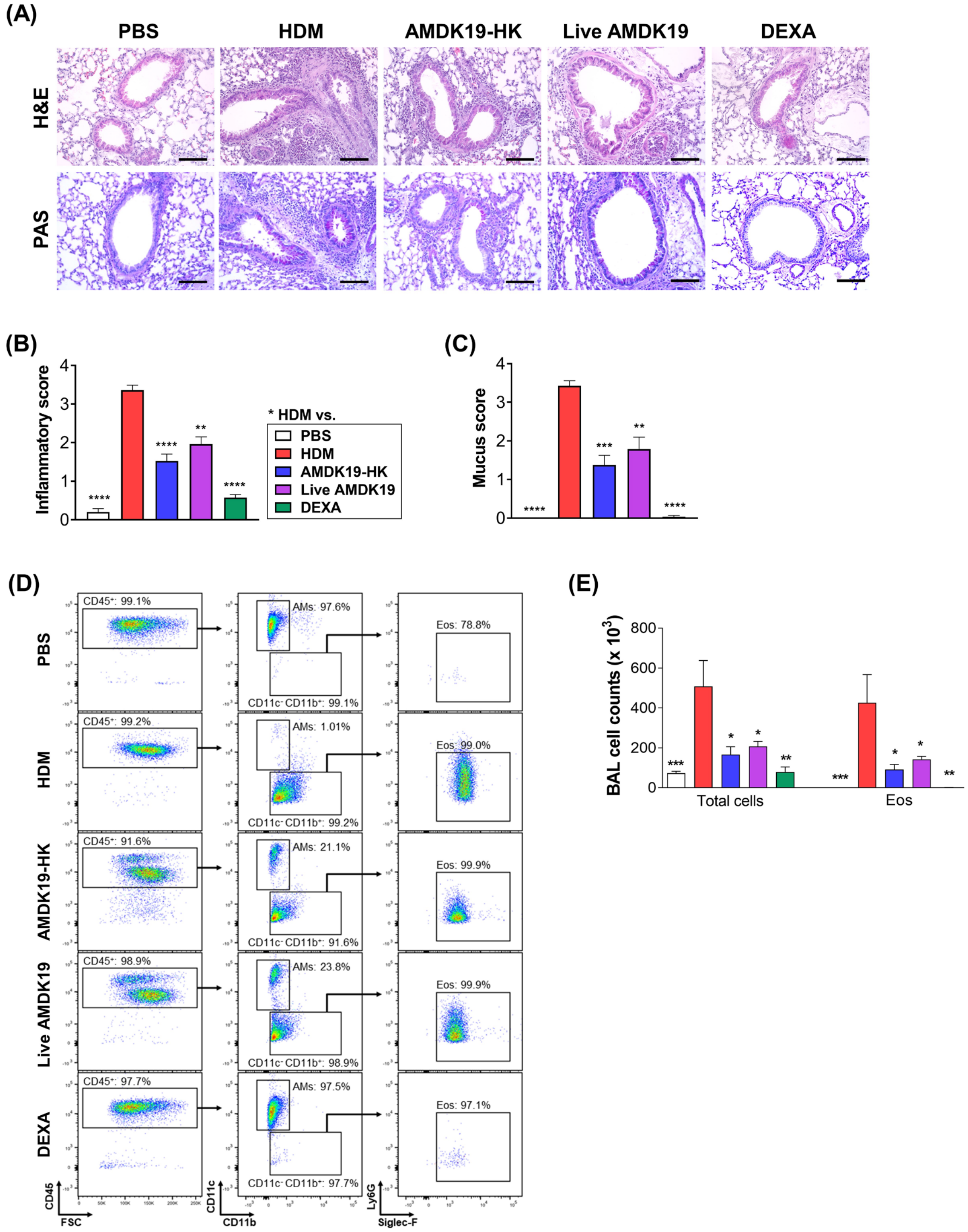

Supplementary Figure S2. Oral administration of pasteurized and live forms of AMDK19 alleviated HDM-induced peribronchial cellular recruitment, goblet cell hyperplasia, and eosinophilia in the airways. (A) A mouse model of AA mice was established as described in Supplementary Figure S1. Mice were given AMDK19 (2.5×10^8 cells/day) via oral gavage from one week before the first sensitization until the end of the experiment. Mice treated with PBS

were used as an untreated control. DEXA (2 mg/kg body weight/day) was used as positive control for treatment. On the end day of the experiment, lungs were collected, fixed, embedded in paraffin, and sectioned at 4-μm thickness. A representative of each group is shown (n = 5 mice/group). (A) H&E-stained slides (upper panel) and PAS-stained slides (lower panel) of each group. Scale bar, 100 μm. (B) For semi-quantification, inflammatory cell infiltration was scored based on 5-point scale (0-4). (C) For semi-quantification, distribution of goblet cells and mucus production were scored based on 5-point scale (0-4). (D) Gating strategy for the identification of BALF leukocytes. Leukocytes were gated as live $CD45^+$ cells. AMs were gated as $CD45^+CD11b^{low}CD11c^+$ cells. Eosinophils were gated as $CD45^+CD11c^{low}CD11b^+Ly6G^{low}Siglec\text{-}F^+$ cells. (E) Count of total leukocytes and eosinophils in BALF. Each dot represents one mouse (n = 5-6 mice per group). Data are expressed as mean ± SEM. * $P < 0.05$, ** $P < 0.01$, *** $P < 0.001$, and **** $P < 0.0001$, Mann–Whitney *U* test. AM, alveolar macrophage; Eos, eosinophil.

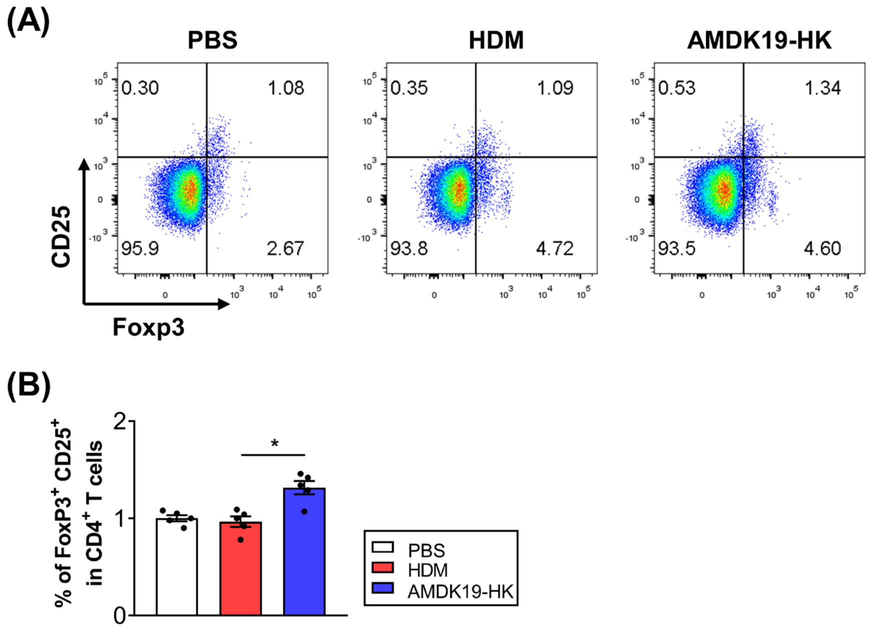

Supplementary Figure S3. AMDK19-HK induced increased frequencies of regulatory T cells in the spleen. A mouse model of AA mice was established by sensitization with HDM extracts and consequent challenge with HDM. AMDK19-HK was given from one week before the first sensitization until the end of the experiment via oral gavage. On the end day of the experiment, the frequency of $CD4^+$ T cells and $FoxP3^+CD25^+$ Treg cells in the spleen. Treg cell frequencies are depicted as the frequency of $FoxP3^+CD25^+$ cells within the CD4 T cell compartment. Data are expressed as mean ± SEM. *$P < 0.05$, Mann–Whitney U test.

1.2 Supplementary Tables

Supplementary Table S1. Primer details used for RT-qPCR

Gene name	Forward primer 5' - 3'	Reverse primer 5' - 3'
Il4	GTCATCCTGCTCTTCTTTCTCG	CTCTCTGTGGTGTTCTTCGTTG
Il5	GGCTTCCTGTCCCTACTCATAA	CTCGCCACACTTCTCTTTTTG
Il13	TCCAGCCTCCCCGATACC	AGCAAAGTCTGATGTGAGAAAGG
Ccl17	CGAGAGTGCTGCCTGGATTACT	GGTCTGCACAGATGAGCTTGCC
Muc5ac	AAAGACACCAGTAGTCACTCAGCAA	TTACTGGAAAGGCCCAAGCA
Muc2	AAACTGCTCTCTGGACTGCC	TTGGTTGGTGTGCTGAGTGT
ZO1	GCCGCTAAGAGCACAGCAA	TCCCCACTCTGAAAATGAGGA
Ocln	TTGAAAGTCCACCTCCTTACAGA	CCGGATAAAAAGAGTACGCTGG
Cldn1	AGGCAACCAGAGCCTTGATGGTAA	CATGCACTTCATGCCAATGGTGGA
Actb	CCCTGAAGTACCCCATTGAAC	CTTTTCACGGTTGGCCTTAG
Gapdh	TGCAGTGCCAGCCTCGT	CAATACGGCCAAATCCG

Supplementary Table S2. Antibodies used for flow cytometry

Antibodies	Source	Identifier
Lung tissues		
Brilliant Violet 510™ anti-mouse CD45 antibody	BD Biosciences	563891
Brilliant Violet 711™ anti-mouse CD103 antibody	BD Biosciences	748255
PE anti-mouse Siglec-F antibody	BD Biosciences	552126
PE/Dazzle™ 594 anti-mouse CD11c antibody	BioLegend	117348
PE/Cyanine7 anti-mouse Ly-6G antibody	BioLegend	127618
Alexa Fluor 647® 450 anti-mouse I-A/I-E antibody	BioLegend	107618
FITC anti-mouse F4/80 antibody	Invitrogen	11-4801-82
eFluor™ 450 anti-mouse CD11b antibody	Invitrogen	48-0112-82
BALFs		
Brilliant Violet 421™ anti-mouse CD45 antibody	BioLegend	103134
APC anti-mouse CD11c antibody	BioLegend	117309
APC/Cyanine7 anti-mouse Ly-6G Antibody	BioLegend	127624
PE anti-mouse Siglec-F antibody	BD Biosciences	552126
FITC anti-mouse/human CD11b antibody	BD Biosciences	553310
Spleen		
PerCP/Cy5.5 anti-CD4 antibody	BioLegend	100540
BV650 anti-CD25 antibody	BioLegend	102038
PE anti-Foxp3 antibody	BioLegend	126404

Supplementary Table S3. Relative abundance of bacteria at phylum, family, and genus levels in cecum contents.

**Microbial features**	**PBS**	**HDM**	**AMDK19-HK**	***P*-value**
**Phyla**				
Firmicutes	67.13 ± 6.28	71.83 ± 5.00	74.10 ± 3.21	0.105
Bacteroidota	31.68 ± 6.06	27.03 ± 4.81	25.16 ± 3.06	0.116
**Families**				
chnospiraceae	45.98 ± 14.83	54.14 ± 3.76	59.95 ± 4.94	0.138
Muribaculaceae	26.31 ± 5.62	20.91 ± 3.22	19.99 ± 3.04	0.123
Lactobacillaceae	5.37 ± 1.66	3.24 ± 1.84	5.82 ± 6.63	0.551
Erysipelotrichaceae	7.66 ± 6.72	6.92 ± 7.08	3.01 ± 1.83	0.618
Rikenellaceae	2.13 ± 0.77	2.41 ± 0.88	2.12 ± 1.12	0.874
Prevotellaceae	1.36 ± 0.57	1.62 ± 0.35	1.76 ± 1.22	0.735
Ruminococcaceae	1.59 ± 0.49	1.35 ± 0.24	1.51 ± 0.87	0.491
Oscillospiraceae	1.92 ± 0.80	2.65 ± 1.01	1.49 ± 0.92	0.334
Bacteroidaceae	1.80 ± 1.14	2.05 ± 1.18	1.21 ± 0.74	0.437
Clostridia_UCG-014	1.64 ± 1.48	1.74 ± 1.50	1.14 ± 0.83	0.794
Clostridiaceae	1.64 ± 1.84	0.17 ± 0.11	0.17 ± 0.07	0.214
**Genera**				
Muribaculaceae	25.87 ± 5.44	20.57 ± 3.21	19.66 ± 2.97	0.123
Lachnospiraceae_NK4A136_group	21.20 ± 10.30	24.77 ± 7.23	35.31 ± 7.59	0.125
Unclassified f__Lachnospiraceae	12.88 ± 3.87	15.45 ± 5.31	14.83 ± 7.61	0.874
Turicibacter	6.60 ± 7.04	1.63 ± 1.33	0.35 ± 0.20	0.138
Lachnospiraceae_UCG-006	5.60 ± 7.41	3.73 ± 2.11	2.90 ± 2.34	0.595
Lactobacillus	5.37 ± 1.66	3.24 ± 1.84	5.82 ± 6.63	0.551
Alistipes	2.13 ± 0.77	2.41 ± 0.88	2.12 ± 1.12	0.874
Bacteroides	1.80 ± 1.14	2.05 ± 1.18	1.21 ± 0.74	0.437
Clostridia_UCG-014	1.64 ± 1.48	1.74 ± 1.50	1.14 ± 0.83	0.794
Clostridium_sensu_stricto_1	1.53 ± 1.87[a]	0.03 ± 0.01[b]	0.05 ± 0.03[ab]	0.019
Lachnoclostridium	1.34 ± 0.50[b]	2.76 ± 0.16[a]	1.29 ± 0.51[b]	0.024
Unclassified f__Oscillospiraceae	1.21 ± 0.46	1.74 ± 0.78	0.95 ± 0.62	0.390
Roseburia	1.01 ± 0.62	2.82 ± 2.48	1.74 ± 0.85	0.390
Faecalibaculum	0.98 ± 0.80	5.17 ± 6.16	2.64 ± 1.94	0.123
[Eubacterium]_xylanophilum_group	0.95 ± 0.20	1.96 ± 1.54	0.89 ± 0.71	0.368
Alloprevotella	0.89 ± 0.64	1.13 ± 0.15	0.99 ± 0.90	0.394

[*] Data were represented by mean ± SD and analyzed by Kruskal–Wallis test with Dunn's post-hoc comparisons test with a significance level of $P < 0.05$.

Supplementary Table S4. Bacteria differentially represented between groups (PBS vs HDM).

<table>
<tr><th>Bacteria</th><th>Enriched group</th><th>LDA score (log 10)</th><th>P-value</th></tr>
<tr><td>p__Firmicutes|c__Clostridia|o__Clostridiales|f__Clostridiaceae|g__Clostridium_sensu_stricto_1</td><td>PBS</td><td>3.955</td><td>0.021</td></tr>
<tr><td>p__Firmicutes|c__Bacilli|o__Erysipelotrichales|f__Erysipelatoclostridiaceae|g__Erysipelatoclostridium</td><td>PBS</td><td>3.340</td><td>0.047</td></tr>
<tr><td>p__Firmicutes|c__Clostridia|o__Lachnospirales|f__Lachnospiraceae|g__Acetatifactor</td><td>PBS</td><td>3.223</td><td>0.037</td></tr>
<tr><td>p__Firmicutes|c__Clostridia|o__Lachnospirales|f__Lachnospiraceae|g__Lachnoclostridium</td><td>HDM</td><td>3.923</td><td>0.021</td></tr>
</table>

Supplementary Table S5. Bacteria differentially represented between groups (HDM vs AMDK19-HK).

<table>
<tr><th>Bacteria</th><th>Enriched group</th><th>LDA score (log 10)</th><th>P-value</th></tr>
<tr><td>p__Firmicutes|c__Clostridia|o__Lachnospirales|f__Lachnospiraceae|g__Lachnoclostridium</td><td>HDM</td><td>3.836</td><td>0.021</td></tr>
<tr><td>p__Firmicutes|c__Bacilli|o__Erysipelotrichales|f__Erysipelotrichaceae|Unclassifiedf__Erysipelotrichaceae</td><td>HDM</td><td>3.124</td><td>0.020</td></tr>
<tr><td>p__Firmicutes|c__Clostridia|o__Lachnospirales|f__Lachnospiraceae|g__Tyzzerella</td><td>HDM</td><td>3.100</td><td>0.043</td></tr>
<tr><td>p__Firmicutes|c__Bacilli|o__Acholeplasmatales</td><td>HDM</td><td>3.093</td><td>0.043</td></tr>
<tr><td>p__Firmicutes|c__Clostridia|o__Lachnospirales|f__Lachnospiraceae|g__Tuzzerella</td><td>HDM</td><td>3.054</td><td>0.047</td></tr>
<tr><td>p__Firmicutes|c__Bacilli|o__Acholeplasmatales|f__Acholeplasmataceae</td><td>HDM</td><td>3.010</td><td>0.043</td></tr>
<tr><td>p__Firmicutes|c__Bacilli|o__Acholeplasmatales|f__Acholeplasmataceae|g__Anaeroplasma</td><td>HDM</td><td>2.994</td><td>0.043</td></tr>
</table>

Supplementary Table S6. Spearman's correlation between *Lachnospiraceae_NK4A136_group* genus and SCFAs in cecal contents.

SCFAs (ng/mg of sample)	*Lachnospiraceae_NK4A136_group*	
	**Coefficient strength**	***P*-value**
Acetate	0.35	0.266
Propionate	0.69	0.0173
Butyrate	0.43	0.161
